# Navigation program: comparison of diagnosis-treatment times among breast cancer patients*

**DOI:** 10.1590/1518-8345.8049.4846

**Published:** 2026-06-15

**Authors:** Fernanda Felipe Pautasso, Maiara Anchau Floriani, Nicole de Moraes Pertile, João Lucas Campos de Oliveira

**Affiliations:** 1 Universidade Federal do Rio Grande do Sul, Escola de Enfermagem, Porto Alegre, RS, Brazil.; 2 Santa Casa de Porto Alegre, Escritório de Valor, Porto Alegre, RS, Brazil.; 3 Santa Casa de Porto Alegre, Escritório da Qualidade e Valor, Porto Alegre, RS, Brazil.

**Keywords:** Nursing, Patient Navigation, Medical Oncology, Breast Cancer, Time-to-Treatment, Nurse’s Role

## Abstract

**Objective:**

to compare diagnostic-treatment time indicators for care provided to women with breast cancer, according to inclusion in a nursing navigation program.

**Method:**

retrospective cohort study conducted at a large hospital. Patients with breast cancer, navigated (n=133) or not (n=226), were included. Sociodemographic and clinical variables were analyzed, and the times (in days) from diagnosis to the start of different treatment modalities constituted the dependent variables. Descriptive and inferential analysis was performed at a significance level of 5%.

**Results:**

patients covered by the Unified Health System had significantly shorter median times between diagnosis and any treatment (77.0) and neoadjuvant chemotherapy (77.0) (p-value < 0.001) than those not covered (100.5 and 112.2, respectively). Patients navigated by supplementary health insurance also had shorter access times to both treatment in general (38.0 versus 47.0; p-value <0.001) and neoadjuvant chemotherapy (31.0 versus 50.0; p-value <0.001). Without considering the health insurance plan, no significant differences were found.

**Conclusion:**

the navigation program had a positive impact on the accessibility of breast cancer diagnosis and treatment. Investment in this type of care management promoted by nurses is recommended, with greater emphasis on the Unified Health System.

## Highlights

**(1)** A reduction in diagnosis-treatment time was observed in patients who received navigation services. **(2)** The effectiveness of the nurse navigator’s work in care was demonstrated. **(3)** The need for investment in the implementation of navigation programs was highlighted.

## Introduction

In early 2024, the International Agency for Research on Cancer (IARC) of the World Health Organization (WHO) released alarming estimates related to the global burden of the disease^1^. Among the statistics, the growing burden of cancer, its disproportionate impact on the most vulnerable populations, and the urgent need to address inequalities related to diagnosis and treatment worldwide stood out^1^ ^-^ ^2^. 

According to the National Cancer Institute (INCA), an estimated 704,000 new cases of cancer will occur in Brazil each year during the 2023-2025 triennium, with the South and Southeast regions accounting for about 70% of the incidence^3^ ^-^ ^4^. Excluding non-melanoma skin cancer, 483,000 new cases are expected, with 49.5% in men and 50.5% in women^4^. The most common type of cancer in women continues to be breast cancer^4^.

Brazil has a high rate of late diagnoses and difficulties in accessing services for the diagnosis and treatment of breast cancer^5^ ^-^ ^6^. Even with political and legal advances to guarantee the rights of cancer patients, such as the approval of Law No. 12,732/2012 (60-day Law) and No. 13,896/2019 (30-day Law) and the National Policy for Cancer Prevention and Control, in practice, there are significant gaps in terms of accessibility, equity, and quality of healthcare for Brazilian women with malignant breast neoplasms^5^ ^,^ ^7^ ^-^ ^9^. 

To improve access to care for women with breast cancer in the Harlem community in New York in the 1990s, patient navigation emerged with the goal of promoting timely access to health systems and services to achieve better clinical outcomes and mitigate the social inequalities that impact such outcomes^10^. Gradually, patient navigation expanded to other countries, such as Canada and Australia, and also to assist with other health conditions^10^. More recently, this care management strategy arrived in Latin America, and in 2019, the first Brazilian breast cancer patient navigation program was reported^11^. In 2023, recognizing the potential of the strategy, the Ministry of Health published, together with the National Policy for Cancer Prevention and Control within the public health system, the National Program for Cancer Patient Navigation^12^. The objective of this legislation is to promote the effective implementation of navigation in the country^12^.

In Brazil, as in the countries where patient navigation originated, the main professional who stands out in this role is the nurse, referred to as the Nurse Navigator (NN)^13^. This professional mobilizes their specialized knowledge, clinical experience, and skills to promote care focused on the needs of patients, family members, and caregivers^14^. Thus, the NN supervises the entire treatment process, empowering patients and acting as a link between them and the entire healthcare system, since their role aims to identify barriers to access of different kinds (navigation needs) and overcome them, achieving more positive results in a timely manner^13^ ^-^ ^14^.

Investments are being made in research to assess the effect of NNs on the clinical outcomes of women with breast cancer, with the aim of strengthening the justification for implementing navigation programs in health organizations and networks^15^ ^-^ ^16^. Studies indicate that women who received navigation services had significantly lower levels of distress, anxiety, and depression during treatment^17^ ^-^ ^18^. A phenomenological study conducted in Peru found that NN support for women with abnormal breast cancer screening results helped overcome barriers to access, optimizing definitive diagnosis and thus accelerating the start of treatment^19^. 

Despite international evidence on the benefits of navigation programs (NP), knowledge about the real impacts of this strategy on healthcare, services, and the lives of patients, including those living with breast cancer, is still in its infancy in Brazil. This lack of knowledge may stem from both the recent implementation of navigation in Brazil and the complexity of the Brazilian healthcare system. In addition, significant regional differences within the country have an impact on access to and quality of healthcare. In this sense, the objective of this study was to compare the diagnostic-treatment time indicators of care provided to women with breast cancer, according to their inclusion in a nursing navigation program.

## Method

### Type of study

Observational study, retrospective cohort type, guided by Strengthening the Reporting of Observational Studies in Epidemiology (STROBE). In this type of study, a population is followed over time with a focus on seeking possible associations between exposure and outcome and, in the case of a retrospective study, past information on the exposure factor is collected^20^. 

### Setting

The survey fields were the Navigation Center and the Outcomes Center, both linked to the Value Office (VO) of a large hospital located in Porto Alegre, RS, Brazil. The Outcomes Center monitors patients in care pathways, including breast cancer. Patient data are collected prospectively and monitored in real time through dashboards, including process analysis, patient clinical profiles, and treatment outcomes over time. The Navigation Center at this institution is responsible for implementing NN in care pathways. It currently consists of an NN who navigates the breast cancer care pathway, structures and implements new NPs, and trains new navigators.

These professional coordinates patient care, accompanying them throughout their treatment journey, from the moment of diagnosis. Their activities include welcoming patients, actively listening, and identifying barriers to care that may compromise their access to timely treatment and follow-up. The NN is responsible for developing and implementing an individualized care plan for each patient, guiding the patient and family in relation to consultations, exams, and therapies, in addition to monitoring treatment adherence and managing possible symptoms resulting from therapeutic interventions.

### Period

Between October 1, 2022, and October 31, 2023. 

### Population

Patients treated in the Breast Cancer Care Line, included in the Outcome Center monitoring database, whether or not accompanied by NP.

### Selection criteria

Inclusion criteria: patients aged 18 years or older, with a confirmed diagnosis of breast cancer who have not undergone previous treatment for the disease, included in the outcome database by June 30, 2023. This deadline for inclusion in the database was set to ensure that all patients in the study had at least three months of outcome follow-up. 

Exclusion criteria: patients who underwent clinical treatment outside the study site, even if they were registered in the database of the survey site; and patients diagnosed with recurrence of the neoplasm at the time of their inclusion in the database.

### Definition of participants

Patients assisted in the Breast Cancer Care Line, included in the Outcome Center monitoring database.

The sample size was calculated using WinPEPI (Software for Epidemiologists for Windows) version 11.65 and based on the study Feasibility of Patient Navigation to Improve Breast Cancer Care in Malaysia^21^. Considering a significance level of 5%, power of 90%, an effect size of navigation of at least 0.5 standard deviations in the time from diagnosis to treatment, and an increase of 20% for possible losses, a minimum total of 204 patients was obtained, with a minimum of 102 in each group.

### Study variables

Inclusion in the NP was considered the comparison factor. The categorical independent variables extracted were: skin color, education level, marital status, residence location, type of health insurance (Unified Health System or other private health insurance), and clinical stage at diagnosis. 

The quantitative variables were: age, time between diagnosis and initiation of any treatment, time between diagnosis and initiation of neoadjuvant treatment, time between diagnosis and surgical treatment, and time between diagnosis and initiation of chemotherapy. Diagnosis-treatment times were considered outcomes (dependent variables), also compared by the navigation factor. The time measurements, in days, were as follows: time between diagnosis and initiation of any treatment, time between diagnosis and neoadjuvant treatment, and time between diagnosis and surgical treatment. 

### Tools used to collect information

The data were collected directly from the Research Electronic Data Capture (REDCap^®^) platform. 

### Data collection

Data collection was performed entirely electronically, based on an institutional computerized system. The variables of interest were transferred from the institutional system via the REDCap^®^ platform to Excel^®^ spreadsheets.

Demographic and clinical variables, including the time between diagnosis and treatment for all patients, were collected by the Outcomes Center team using specific forms and subsequently entered into the institutional database via the REDCap^®^ platform. The forms were developed by the sector team based on the criteria of the International Consortium for Health Outcome Measurement (ICHOM) for patient-centered outcome measures for non-metastatic breast cancer. 

Data collection is performed through review of medical records and telephone contact with patients at the following times: before the start of treatment, six months and one year after treatment. Patients are followed annually for up to 10 years.

Data related to the navigation program were collected by the nurse navigator upon inclusion in the program and at the time of each patient visit and entered prospectively into a specific form developed by the Navigation Center team in the institutional database via the REDCap^®^ platform. Patients are followed up in the NP according to their navigation needs, for as long as they require support from the NN, with no time limit, even after completing their main treatments.

### Data processing and analysis

The data were organized in an Excel^®^ spreadsheet and imported into the Statistical Package for the Social Sciences (SPSS) software, version 29.0. The Kolmogorov-Smirnov test was used to assess the normality of distribution. Descriptive and inferential analyses were performed at a significance level of 5%. To characterize the participants, measures of central tendency (median and interquartile range) and absolute and relative frequencies of categorical variables were described. The sample was matched considering the age variable to reduce possible confounding biases between groups. 

The association of sociodemographic and clinical variables with the navigation factor was verified using Pearson’s chi-square test. Given the asymmetric distribution, the comparison of variables referring to the time between diagnosis and types of treatment of patients included or not included in the navigation program was verified using the Mann-Whitney test. 

To mitigate potentially confounding variables, the same comparison was performed to assess the difference in times by segmenting the group of patients by type of health insurance (Unified Health System or Supplementary Health), adding the navigation factor. Differences with p≤0.05 were considered statistically significant.

### Ethical aspects 

The research complied with current ethical legislation, in accordance with the terms of Resolution No. 466/2012 of the National Health Council. It was submitted to the Research Ethics Committee of the study institution and carried out, after approval, under opinion No. 6,620,728/2024. All participants were provided with a free and informed consent form, sent electronically via the WhatsApp^®^ application, through the Google Forms^®^ platform.

## Results

A total of 359 women diagnosed with breast cancer and without prior treatment were included in the care pathway. The average follow-up time for patients included in the NP was 10 months.

Of the total sample, 37% (n=133) were included in the NP; 46% (n=165) were from Porto Alegre, and 64% (n=194) were from Greater Porto Alegre and other municipalities in Rio Grande do Sul. 

The mean age at diagnosis for the entire sample was 58.6±13.6 years. When comparing the groups of non-navigated patients (60.4±13.8 years) and navigated patients (55.5±12.7 years), there was no statistical difference (p=0.232).

Among the navigated patients, significantly higher percentages were found for the variables non-white skin color, stage III and IV at diagnosis, and chemotherapy treatment type. In the health insurance type variable, it was observed that most of the patients included in the NP (93.2%) were assisted by the SUS, and among those not included were patients assisted by the supplementary health system (63.7%) (Table 1).

**Table 1:** Demographic and clinical characteristics of breast cancer patients, according to inclusion in the navigation program (n = 359). Porto Alegre, RS, Brazil, 2023

Variable	Total patients	Patients included in the navigation program	Patients not included in the navigation program	p-value*
	n (%)	n (%)	n (%)	
Health Insurance				
Unified Health System	206 (57.4%)	124 (93.2%)	82 (36.3%)	<0.001
Supplementary health insurance	152 (42.6%)	9 (6.8%)	144 (63.7%)	
Skin color				
White	301 (86%)	101 (77.1%)	200 (91.3%)	<0.001
Non-white	49 (14%)	30 (22.9%)	19 (8.7%)	
Education				
Incomplete elementary education	84 (24.7%)	19 (35.3%)	42 (19%)	0.001
Complete elementary education or higher	256 (75.3%)	77 (64.7%)	179 (81%)	
Marital status				
Married or with partner	148 (42.4%)	48 (37.8%)	100 (45%)	0.228
Without partner	201 (57.6%)	79 (62.2%)	122 (55%)	
Staging at diagnosis				
0, I or II	227 (79.4%)	77 (64.7%)	150 (89.8%)	<0.001
III or IV	59 (20.6%)	42 (35.3%)	17 (10.2%)	
Type of treatment				
Surgical	283 (78%)	74 (55.6%)	209 (92.5%)	<0.001
Chemotherapy	150 (41.8%)	98 (73.7%)	52 (23.3%)	<0.001
Hormone therapy	117 (32.6%)	40 (30.1%)	77 (34.1%)	0.507
Radiotherapy	149 (41.5%)	46 (34.6%)	103 (45.6%)	0.054
Number of treatments				
Only 1	168 (46.9%)	66 (49.6%)	102 (45.3%)	0.499
>1	190 (53.1%)	67 (50.4%)	123 (54.7%)	0.499

*Pearson’s chi-square test

With regard to the interval between diagnosis and the start of neoadjuvant treatment, as well as between diagnosis and the start of any treatment (Table 2), no statistically significant differences were identified between the groups, even though the time was longer among patients included in the NP compared to those not included. On the other hand, regarding the interval between diagnosis and the start of surgical treatment, it was observed that, in navigated patients, the time was significantly longer than in non-navigated patients.

Furthermore, regarding the diagnosis-surgical treatment interval for the general group, the median time between diagnosis and treatment was 94.0 days, with interquartile ranges varying from -19 to 652 days. This is due to the fact that some patients initially diagnosed with benign anatomopathological findings may have malignancy confirmed only after surgery. In these cases, the record of the start of treatment precedes definitive histopathological confirmation, generating negative intervals between diagnosis and the start of treatment in the database.

**Table 2:** Comparison of time between diagnosis and treatment modalities among breast cancer patients included or not included in a navigation program (n = 359). Porto Alegre, RS, Brazil, 2023

Variable	Patients included in the navigation program	Patients not included in the program	Total patients	p-value*
	Median + Interquartile ranges	Median + Interquartile ranges	Median + Interquartile ranges	
	(days)	(days)	(days)	
	Median	Median	Median	
Time between diagnosis and start of any treatment (P25/75)	75.0 (50.0 / 98.0)	69.0 (42.0 / 112.0)	72.0 (0 / 271.0)	0.394
Time between diagnosis and neoadjuvant treatment (P25/75)	72.0 (48.0 / 98.5)	56.0 (45.5 / 99.0)	71.5 (17.0 / 211.0)	0.273
Time between diagnosis and surgical treatment (P25/75)	231.0 (79.5 / 284.7)	79.0 (42.0 / 147.2)	94.0 (-19 / 652)	<0.001

*Mann-Whitney test

Regarding diagnosis-treatment times in the group of patients treated by the Unified Health System (SUS) (Table 3), the median time between diagnosis and any treatment was 84 days. When considering the navigation factor, navigated patients had median times between diagnosis and any treatment (77 days) and neoadjuvant chemotherapy treatment (77 days) that were significantly shorter than those not included in the NP. However, in relation to the time between diagnosis and the start of surgical treatment, navigated patients had a significantly longer interval than those not included in the NP.

**Table 3:** Comparison of time between diagnosis and treatment modalities among breast cancer patients treated by the Unified Health System, whether or not included in a navigation program (n = 153). Porto Alegre, RS, Brazil, 2023

Variable	Patients included in the navigation program	Patients not included in the program	Total number of patients	p-value*
	Median + Interquartile ranges	Median + Interquartile ranges	Median + Interquartile ranges	
	(days)	(days)	(days)	
	Median	Median	Median	
Time between diagnosis and start of any treatment (P25/75)	77.0 (55.0 / 100.0)	100.5 (73.2 / 150.2)	84.0 (60.0 / 122.0)	<0.001
Time between diagnosis and neoadjuvant treatment (P25/75)	77.0 (53.0 / 99.7)	85.5 (50.7 / 112.2)	77.0 (52.7 / 101.0)	<0.001
Time between diagnosis and surgical treatment (P25/75)	236.0 (85.0 / 285.0)	128.0 (80.0 / 191.5)	152.0 (81.0 / 264.7)	<0.001

In the group of patients covered by supplementary health insurance (Table 4), the median time between diagnosis and any treatment was 42.5 days. When considering inclusion or exclusion from the navigation program, navigated patients had significantly shorter times between diagnosis and initiation of any treatment (38 days) and diagnosis and initiation of neoadjuvant chemotherapy (31 days) than non-navigated patients, who had intervals of 47 and 50 days, respectively.

**Table 4:** Comparison of time between diagnosis and treatment modalities among breast cancer patients covered by supplementary health insurance, whether or not included in a navigation program (n = 206). Porto Alegre, RS, Brazil, 2023

Variable	Patients included in the navigation program	Patients not included in the program	Total patients	p-value*
	Median + Interquartile ranges	Median + Interquartile ranges	Median + Interquartile ranges	
	(days)	(days)	(days)	
	Median	Median	Median	
Time between diagnosis and start of any treatment (P25/75)	38.0 (30.5 / 46.5)	47.0 (28.0 / 62.0)	42.5 (28.2 / 60.5)	<0.001
Time between diagnosis and neoadjuvant treatment (P25/75)	31.0 (30.5 / 41.5)	50.0 (17.0 / 56.0)	35.5 (28.5 / 51.5)	<0.001
Time between diagnosis and surgical treatment (P25/75)	55.0 (44.0 / 264.0)	49.0 (29.0 / 81.0)	77.0 (52.5 / 101.0)	<0.001

*Mann-Whitney test

Regarding the time between diagnosis and the start of surgical treatment, the variable showed a significantly longer interval in navigated patients (p<0.05). When considering inclusion or exclusion in the navigation program, segmented by type of health insurance, it was observed that the time between diagnosis and surgical treatment remained longer in this group.

## Discussion

The results obtained in this study corroborate the epidemiological profile frequently described in the literature for the breast cancer population. The mean age of the sample was 58.6±13.6 years, and there was no difference between the groups. The study that analyzed the epidemiological profile of people diagnosed with breast cancer in Brazil showed that the incidence of the disease in the 50-59 age group is predominant^4^. The same study also pointed out that 98.7% of cases occur in women. According to data from INCA, female breast cancer is predominant in all regions of Brazil, with cases of the disease in men considered rare^4^. 

Non-white skin color, incomplete elementary school education, and care through the SUS were more frequent in the group included in the NP. Socioeconomic factors such as unemployment, low educational level, poverty, and skin color/race/ethnicity are among the most important social determinants of health, including in oncology^21^ ^-^ ^22^. Clinical aspects, demographic characteristics, and other ethnic inequalities in access to health services have been identified as factors influencing access to timely diagnosis and treatment of breast cancer in women^23^ ^-^ ^24^. Thus, in order to reduce disparities in access, patient navigation is justified, as confirmed by the findings of this study regarding the best indicators of time in the modalities of diagnosis and any treatment, and diagnosis and neoadjuvant treatment among patients treated by both the SUS and private health insurance.

Patient navigation aims to address disparities in access to healthcare and promote timely movement of individuals through the healthcare system^13^ ^-^ ^14^ ^,^ ^24^. It can be inferred that the group of navigated patients, when segmented by care modality/health plan, especially at the time of diagnosis and initiation of any treatment and diagnosis of neoadjuvant treatment, obtained better management of barriers to accessing care, given the better performance among these indicators. The approach taken by the navigator provides patients with constant support and communication, providing essential information about the diagnosis and therapeutic journey and helping to maintain better adherence to treatment^25^. Therefore, this study is in line with the premises of patient navigation.

Regarding staging at diagnosis, 74.5% of the sample had neoplasia in stages 0, I, and II, with no difference between the groups. Among the patients included in the navigation program, there was a higher volume of stage III and IV diagnoses (35.3%) compared to patients not included (10.2%). This can be explained by the fact that the patients treated by the SUS corresponded mainly to the “navigated” group. In other words, although navigation improved the time to access neoadjuvant treatment and any treatment, patients in the public system still took much longer to access treatment than those in the supplementary system, which may have resulted in more advanced cancer staging. Although this reality corroborates the social inequalities in Brazil, SUS patients who were included in the NP had significantly better results in terms of access time to clinical treatment than those who were not. This suggests the need for government investment in this type of nursing practice, especially in progressive diseases.

A cross-sectional, analytical, retrospective study conducted in 2024 at a public hospital in Brasília found that 35% of patients were diagnosed with stage III disease, demonstrating later diagnosis in these women^26^. Another study, conducted in Rio de Janeiro, identified a prevalence of 43.7% of diagnoses at an advanced stage (stages III and IV)^25^. This condition was observed among women with a mean age of 49 years, non-white skin color, who lived without a partner and were referred through the SUS^27^. Although to a lesser extent than the studies cited, our findings show that there is room for improvement in reducing diagnoses in stages III and IV, regardless of whether patients are included in the patient navigation program. 

The management of breast cancer requires a personalized approach, taking into account, among other factors, the staging of the disease, immunohistochemistry results, and tumor size^28^. Currently, surgical intervention to remove the tumor is no longer the only critical step in treatment, but rather part of a broader process faced by women^28^ ^-^ ^29^. Chemotherapy, radiotherapy, hormone therapy, and immunotherapy, for example, are complementary forms of treatment that, over time, have contributed significantly to increasing survival rates and the period free of disease progression^29^. Considering the number of treatment modalities performed, in the entire sample, 53.1% of patients underwent more than one treatment, with no significant difference between navigated and non-navigated patients. The data suggest that there are several treatment modalities available in the service surveyed, regardless of whether or not support is provided by the NN.

Regarding the types of treatment performed, chemotherapy (73.7%) predominated among navigated patients. Patients undergoing chemotherapy may experience various adverse effects and remain in treatment for several months, depending on the therapy indicated, which can hinder follow-up or even lead to treatment discontinuation^30^ ^-^ ^32^. The signs and symptoms of chemotherapy treatment, such as nausea, vomiting, fatigue, mucositis, and febrile neutropenia, are some of the side effects that contribute to this^30^ ^-^ ^32^. Adherence to treatment, in this sense, is a multidimensional process that is influenced by social, economic, and individual aspects of the person and requires the help of health professionals trained in the health education process^30^ ^-^ ^32^. Thus, the coordination of care performed by the NN is focused on ensuring better adherence to treatment and reducing dropout rates due to these side effects.

Among patients who did not undergo navigation, there was a predominance of women who underwent surgical procedures (92.5%). This can be attributed to the fact that patients who undergo only this type of treatment do not follow clinical treatment at the service, so they are not included in the NP, and one of the criteria for inclusion in the NP is to undergo all treatment at the service. Another hypothesis for this finding is the higher concentration of non-navigated patients in the supplementary health group, which had shorter access times to surgical treatment and, consequently, may not have needed any other type of care besides surgery.

In Brazil, Law No. 12,732/2012, enacted by the Ministry of Health (MS), established a 60-day deadline for initiating treatment of patients diagnosed with malignant neoplasia, counted from the confirmation of diagnosis by an anatomopathological examination report to the first day of the indicated therapeutic modality^8^. Research indicates that this interval, when longer than 60 days, may be associated with a worse prognosis^33^ ^-^ ^34^. A study conducted in the United States evaluated the time between diagnosis and treatment of low-income women with breast cancer and found that about 10% wait an interval greater than or equal to 60 days to start treatment^33^. This factor is associated with a 66% decrease in overall 5-year survival in this group^33^. 

The results of this study show that, despite being close to the legal recommendation^8^, the time between diagnosis and any treatment and between diagnosis and the start of neoadjuvant treatment at the research site still requires action to improve this interval, especially in patients in the public health system. This reality reaffirms that the SUS requires significant and permanent investment, as well as reinforcing a critical view of the scope of its premise of comprehensiveness, including the optimization of access time. As the results described above attest, the performance of the NN may be a promising strategy for this purpose. 

In the analysis of the variable time between diagnosis and the start of surgical treatment, it was evident that mainly patients treated by the SUS had an extended interval for its performance. This finding may be associated with the fact that, in the analysis of this variable, not only surgical procedures as first treatment were considered, but also procedures performed after completion of neoadjuvant systemic treatment (chemotherapy or hormone therapy) were included in this result. This is explained by the fact that, when the date of surgery was entered into the database, it was not specified whether it was before or after neoadjuvant systemic treatment, making it impossible to select this variable for a more accurate analysis. 

Neoadjuvant therapy refers to systemic treatment administered prior to treatment considered to have the greatest curative impact, usually surgery^35^ ^-^ ^36^, and lasts an average of 4 to 6 months in breast cancer, depending on the therapeutic protocol indicated^37^. Previously, it was reserved for patients with locally advanced or inoperable tumors and was intended only to reduce the size of the tumor to allow for less extensive and mutilating surgery^35^ ^-^ ^36^. However, currently, more patients with early-stage breast cancer are indicated for this type of treatment^35^ ^-^ ^36^. Patients with early-stage disease may be candidates for neoadjuvant therapy due to potential benefits in predictive and prognostic variables. Furthermore, this type of treatment has provided the opportunity to perform more conservative surgeries in order to reduce the need for total mastectomies^35^. Thus, the extended interval until surgical treatment may be linked to the volume of post-neoadjuvant surgeries, considering that 53% of patients undergo more than one treatment and the staging at diagnosis, since a significantly higher percentage of patients in the sample (89.8%) had early stages at diagnosis.

When analyzing the general group of patients, without segmentation by health insurance plan, it was observed that there was no significant difference between the measured access time indicators. Patients who received navigation services had longer access times to treatment. However, it is important to note that the type of health insurance plan influenced this finding, given that the diagnosis-treatment time indicators for women covered by private health insurance are much lower than those covered by the SUS, regardless of whether or not they were assisted by a nurse navigator. This confirms that social inequalities are a marker in access to breast cancer care^4^ ^-^ ^6^; and, considering the results segmented by type of health insurance, nursing navigation may be a fruitful strategy for addressing these inequalities. 

The findings related to diagnosis and treatment times reinforce the worrying disparity, especially among patients treated by the SUS, since this portion of the sample contributed to the increase in the time to access care. Given this, it is essential to ensure that patients in the public health system have access to cancer screening and early diagnosis programs, enabling more agile contact with the NN, which may positively impact the time to access care.

A Brazilian study, based on the analysis of data collected from DATASUS and *PAINEL-Oncologia*, showed that 43.6% of individuals diagnosed with breast cancer by the SUS had intervals between diagnosis and treatment of more than 60 days. Furthermore, only 21.2% started treatment between 31 and 60 days^37^. The cross-sectional study conducted in Piauí showed that, between 2016 and 2017, 71% of women diagnosed with breast cancer had a median interval of 83.8 days to start the indicated oncological treatment^38^. The authors attributed this delay mainly to a lack of coordination in the oncology care network and suggested that there is a need to coordinate flows and organize specialized care points^38^. Nurses play a key role in this process, as they naturally promote care management, which can contribute to the coordination of care at the health network’s points of care. 

The retrospective cohort study conducted in Minas Gerais showed a median time of 71.5 days between diagnosis and treatment in women treated in the public system and pointed out that factors related to social inequalities may influence access to breast cancer care^39^. The findings of this study are consistent with those reported in the literature.

Regarding patients in the supplementary system, it was also evident that navigated patients had significantly shorter median times from diagnosis to any treatment and from diagnosis to neoadjuvant treatment than non-navigated patients. This is interesting and demonstrates that navigation is beneficial regardless of the healthcare plan. In the case of supplementary health care, in addition to improving clinical outcomes, NN can contribute to the profitability and financial sustainability of services, since timely care is a factor in this regard. 

The experience report described the work of NNs in caring for breast cancer patients in a private hospital in a municipality in the south of the country and demonstrated that, through actions carried out by the professionals, it was possible to ensure that patients included in the service’s NP received their treatment in a timely manner^40^. In addition, the systematic review led by Korean researchers indicated that the studies (n=6) included showed that nurse navigators reduced the number of days between diagnosis and treatment by approximately 17.5 days^41^. Such evidence adds weight to the findings of this study, confirming that NN, through care coordination, mitigation of access barriers, and guidance on diagnosis and treatment journey, is capable of improving access to health care, even in situations where time is as important as cancer. 

Another recently published study showed that, after implementing a navigation program for breast cancer patients in southern Brazil, it was possible to reduce the time between diagnosis and the start of neoadjuvant treatment by approximately 26%^42^. The significantly shorter time intervals (diagnosis-neoadjuvant treatment and diagnosis-any treatment) presented by patients included in the NP compared to those not included reached a difference of 23 days in those treated in the public system and 29 days in the private system. Delays in starting cancer treatment are known to seriously compromise clinical outcomes. Prolonged delays in the diagnosis-to-treatment interval are directly associated with lower survival rates for breast cancer patients^43^, justifying efforts to mitigate and resolve barriers that may interfere with this indicator. This demonstrates that the findings corroborate what has been evidenced in scientific publications, proving that, through nursing navigation, it is possible to positively impact the times considered critical for obtaining the best clinical outcomes.

It is prudent to assume that the limitations of this study include the absence of management of other potentially confounding variables besides the type of health insurance plan, and the natural limitation of possible inconsistency in records. More robust analyses considering other outcomes besides time to treatment access are highly recommended. However, the study is relevant and provides scientific legitimacy to support the dissemination of patient navigation coordinated by nurses. 

This study makes a relevant contribution to the advancement of knowledge by demonstrating, through the comparison of diagnosis-treatment times, the potential effectiveness of a navigation program for breast cancer patients. Thus, this research highlights the importance of navigation as a strategy capable of optimizing access to oncological care, minimizing barriers to care in the health system, and potentially improving clinical outcomes. The data presented can potentially support the implementation of health policies focused on reducing delays in cancer treatment.

## Conclusion

Breast cancer patients included in a nurse-led navigation program had significantly shorter wait times for treatment in general and neoadjuvant chemotherapy than patients who were not included in the program. This was particularly evident when the navigated and non-navigated patient groups were segmented by type of health insurance (public or private). In both cases, navigated patients had significantly better times, although patients from the public system still took significantly longer to access care. 

Strategies developed by the NN in the NP, such as assessing barriers to access to care, developing and implementing an individualized care plan, and conducting health education activities aimed at patients and family members regarding diagnosis and the treatment journey, can contribute positively to these critical intervals. Therefore, in addition to highlighting the effectiveness of the Nurse Navigator’s work, this study attests to the need to invest in the implementation of navigation in health services and in breast cancer screening and early diagnosis programs in the SUS. It is important to note that future research, especially prospective and controlled studies, needs to be conducted to evaluate broader outcomes, such as survival, quality of therapeutic adherence, and quality of life, in order to strengthen the evidence on the effectiveness of nursing navigation in cancer care.

## Data Availability

All data generated or analysed during this study are included in this published article.

## References

[1] Organização Pan-Americana da Saúde (2024). Carga global de câncer aumenta em meio à crescente necessidade de serviços.

[2] World Health Organization (2023). Universal health coverage (UHC).

[3] Instituto Nacional de Câncer José Alencar Gomes da Silva (2022). Estimativa 2023: incidência de câncer no Brasil.

[4] Santos MO, Lima FCS, Martins LFL, Oliveira JFP, Almeida LM, Cancela MC (2023). Estimated cancer incidence in Brazil, 2023-2025. Rev Bras Cancerol.

[5] Almeida A (2023). Panorama da atenção ao câncer de mama no SUS.

[6] Guckert LE, Borne WWM, Dinkoski JS, Maran LG, Peruci C, Bonfanti MM (2025). Challenges and proposals for the early diagnosis of breast cancer in women under 50 years in Brazil. REAC.

[7] Azriful A, Mallapiang F, Kurniati Y (2021). Literature review: social determinant of health in breast cancer patients survival. Open Access Maced J Med Sci [Internet].

[8] Brasil (2012). Lei nº 12.732, de 22 de novembro de 2012. Dispõe sobre o primeiro tratamento de paciente com neoplasia maligna comprovada e estabelece prazo para seu início. Diário Oficial da União.

[9] Brasil (2019). Lei nº 13.896, de 30 de outubro de 2019. Altera a Lei nº 12.732, de 22 de novembro de 2012. Diário Oficial da União.

[10] Freeman HP, Rodriguez RL (2011). History and principles of patient navigation. Cancer.

[11] Rohsig V, Silva P, Teixeira R, Lorenzini E, Maestri R, Saraiva T (2019). Nurse navigation program outcomes from a breast cancer center in Brazil. Clin J Oncol Nurs.

[12] Brasil (2023). Lei nº 14.758, de 19 de dezembro de 2023. Institui a Política Nacional de Prevenção e Controle do Câncer no âmbito do Sistema Único de Saúde (SUS). Diário Oficial da União.

[13] Maia GKC, Ramos RS, Pimentel NBL, Padilha RA, Peres EM, Pinheiro APB, Andrade KBS, Carvalho EC, Souza NVDO, Varella TCMML, Soares SSS (2024). Saberes sobre o cuidado de enfermagem direcionado ao paciente oncológico: contribuições para a prática clínica.

[14] Pautasso FF, Lobo TC, Flores CD, Caregnato RCA (2020). Nurse navigator: development of a program for Brazil. Rev. Latino-Am. Enfermagem.

[15] Bidstrup PE, Johansen C, Kroman N, Belmonte F, Duriaud HM, Dalton SO (2023). Effect of a nurse navigation intervention on mental symptoms in patients with psychological vulnerability and breast câncer: The REBECCA randomized clinical trial. JAMA Netw Open.

[16] Rajabiun S, Cabral HJ, Chen CA, Lloyd-Travaglini C, Dugas JN, Amburgey D (2025). Cost and activity analysis for a citywide patient navigation intervention to engage underserved patients in breast cancer treatment. Cancer.

[17] Mertz BG, Dunn-Henriksen AK, Kroman N, Johansen C, Andersen KG, Andersson M (2017). The effects of individually tailored nurse navigation for patients with newly diagnosed breast cancer: a randomized pilot study. Acta Oncol.

[18] Loiselle CG, Attieh S, Cook E, Tardif L, Allard M, Rousseau C (2020). The nurse pivot-navigator associated with more positive cancer care experiences and higher patient satisfaction. Can Oncol Nurs J.

[19] Matassini-Eyzaguirre SM, Figueroa-Montes LE (2021). Navegación de pacientes con sospecha de cáncer de mama: un estudio cualitativo en Lima, Perú. Rev Cuerpo Med HNAAA.

[20] Camargo LMA, Silva RPM, Meneguetti DUO (2019). Research methodology topics: cohort studies. J Hum Growth Dev.

[21] Yeoh ZY, Jaganathan M, Rajaram N, Rawat S, Tajudeen NA, Rahim N (2018). Feasibility of patient navigation to improve breast cancer care in Malaysia. J Glob Oncol.

[22] Handa LYS, Paiva LS, Sousa LVA (2025). Epidemiological analysis of breast cancer screening and treatment protocols in the Brazilian context. Clin Oncol Lett.

[23] Silva DM, Cavalcante YA, Oliveira BLCA, Lopes MVO, Fernandes AFC, Pinheiro AKB (2025). Social health determinants associated with mammography performance according to the National Health Survey. Cien Saude Colet.

[24] Goulart MGS, Silva MR, Dias KB (2025). The impact of nurse navigator on oncology patient care. Rev Saude Desenvolv.

[25] Moraes MAC, Assis MCS, Acosta AM, Fernandes APW, Trevisan BF, Lorenzzoni AMV (2025). Effectiveness of the navigation of cancer chemoradiotherapy patients: a retrospective cohort study. Rev Gaucha Enferm.

[26] Britto CHMG, Siqueira F, Mascarenhas ECP (2024). Epidemiological profile of patients treated in a public service with Breast Cancer in Brasilia. Health Resid J.

[27] Santos TB, Borges AKM, Ferreira JD, Meira KC, Souza MC, Guimarães RM (2022). Prevalence and factors associated to advanced stage breast cancer diagnosis. Cien Saude Colet.

[28] Maroun PS, Gomes R, Silva A (2024). Breast cancer cultural representations: a scoping review. Cien Saude Colet.

[29] Zuqui R, Oliveira VN, Barreto SN, Almeida JRB, Costa ACMSF, Romeiro ET (2023). Evolution of cancer treatment targeted therapies and immunotherapy. Rev Ibero-Am Hum Cienc Educação.

[30] Pimentel MSSM, Macêdo ABPC, Costa SC, Ávila JMN, Véras RFO, Mendes RA (2024). Adverse effects on neoadjuvant chemotherapy in patients with breast cancer. Rev Ibero-Am Hum Cienc Educação.

[31] Sousa FVS, Borges APS, Ribeiro MA (2025). Predictors of non-adherence and non-persistence to adjuvant hormonal therapy in women with breast cancer: a systematic review. Rev Bras Cancerol.

[32] Leite GC, Ruhnke BF, Valejo FAM (2021). Correlation between time of diagnosis, treatment and survival in patients with breast câncer: a literature review. Colloq Vitae.

[33] McLaughlin JM, Anderson RT, Ferketich AK, Seiber EE, Balkrishnan R, Paskett ED (2012). Effect on survival of longer intervals between diagnosis and treatment initiation among low-income women with breast cancer. J Clin Oncol.

[34] Jomar RT, Velasco NS, Mendes GLQ, Guimarães RM, Fonseca VAO, Meira KC (2023). Factors associated with time-to-treatment initiation of breast cancer. Cien Saude Colet.

[35] Mendes LF, Oliveira HF, Lucena LN, Mendes NVO (2025). Tratamento neoadjuvante em câncer de mama: uma revisão sistemática. Cienc Life.

[36] Ministério da Saúde (2024). Protocolo clínico e diretrizes terapêuticas: câncer de mama.

[37] Nogueira MC, Atty ATM, Tomazelli J, Jardim BC, Bustamante-Teixeira MT, Azevedo e Silva G (2023). Frequency and factors associated with delay in breast cancer treatment in Brazil. Epidemiol Serv Saude.

[38] Sousa SMMT, Carvalho MGFM, Santos LA, Mariano SBC (2019). Access to treatment of women with breast cancer. Saude Debate.

[39] Campos AAL, Guerra MR, Fayer VA, Ervilha RR, Cintra JRD, Medeiros IR (2022). Time to diagnosis and treatment for breast cancer in public and private health services. Rev Gaucha Enferm.

[40] Osorio AP, Flôr JS, Saraiva TKG, Maestri RN, Rohsig V, Caleffi M (2020). Nursing navigation in breast cancer care during the pandemic: an experience report. J Nurs Health.

[41] Oh J, Ahn S (2021). Effects of nurse navigators during the transition from cancer screening to the first treatment phase: a systematic review and meta-analysis. Asian Nurs Res.

[42] Pautasso FF, Trevilato DD, Caregnato RCA, Floriani MA, Pertile NM, Junior Dal Pizzol A (2024). Trajectory for implementation of a patient navigation program in oncology: experience report. Online Braz J Nurs.

[43] Zhu S, Li S, Huang J, Fei X, Shen K, Chen X (2023). Time interval between breast cancer diagnosis and surgery is associated with disease outcome. Sci Rep.

