# Supplementary material for: Navigation program: comparison of diagnosis-treatment times among breast cancer patients
Source: Rev Lat Am Enfermagem. 2026 Jun 15;34:e4846. doi: 10.1590/1518-8345.8049.4846 (PMC13277357; doi:10.1590/1518-8345.8049.4846)
Supplement: Translation

# Translation: Programa de navegação: comparação dos tempos diagnóstico-tratamento entre pacientes com câncer de mama*

**DOI:** 10.1590/1518-8345.8049.4847

**Authors:** Fernanda Felipe Pautasso, Maiara Anchau Floriani, Nicole de Moraes Pertile, João Lucas Campos de Oliveira

**Affiliations:** 1 Universidade Federal do Rio Grande do Sul, Escola de Enfermagem, Porto Alegre, RS, Brasil.; 2 Santa Casa de Porto Alegre, Escritório de Valor, Porto Alegre, RS, Brasil.; 3 Santa Casa de Porto Alegre, Escritório da Qualidade e Valor, Porto Alegre, RS, Brasil.

**Keywords:** Enfermagem, Navegação de Pacientes, Oncologia, Câncer de Mama, Tempo para o Tratamento, Papel do Profissional de Enfermagem

## Abstract

**Objetivo:**

comparar os indicadores de tempo diagnóstico-tratamento do cuidado prestado às mulheres com câncer de mama, conforme inclusão em um programa de navegação em enfermagem.

**Método:**

coorte retrospectiva, realizada em um hospital de grande porte. Foram incluídas pacientes com câncer de mama, navegadas (n=133) ou não (n=226). Analisou-se variáveis sociodemográficas e clínicas; e os tempos (em dias) de diagnóstico e início de diferentes modalidades de tratamento constituíram as variáveis dependentes. Realizada análise descritiva e inferencial, em nível de significância de 5%.

**Resultados:**

pacientes navegadas atendidas pelo Sistema Único de Saúde apresentaram tempos medianos entre diagnóstico e qualquer tratamento (77,0) e quimioterapia neoadjuvante (77,0) significativamente menores (p-valor<0,001) do que as não incluídas (100,5 e 112,2, respectivamente). Pacientes navegadas conveniadas à saúde suplementar também apresentaram menores tempos de acesso tanto ao tratamento em geral (38,0 *versus* 47,0; p-valor<0,001) como ao quimioterápico neoadjuvante (31,0 *versus* 50,0; p-valor<0,001). Sem considerar o convênio de atendimento, não foram verificadas diferenças significativas.

**Conclusão:**

o programa de navegação impactou positivamente na acessibilidade diagnóstico-tratamento do câncer de mama. Recomenda-se investimento nesta modalidade de gerenciamento do cuidado promovida pelo enfermeiro, com maior ênfase no Sistema Único de Saúde.

## Destaques

**(1)** Evidenciou-se redução do tempo diagnóstico-tratamento nas pacientes navegadas. **(2)** Evidenciou-se a efetividade do trabalho do enfermeiro navegador no cuidado. **(3)** Aponta-se a necessidade de investimento na implementação de programas de navegação.

## Introdução

No início de 2024, a Agência Internacional de Pesquisa sobre o Câncer (IARC) da Organização Mundial da Saúde (OMS) divulgou estimativas preocupantes relacionadas à expressão global da doença^1^. Entre as estatísticas, destacou-se o crescente ônus do câncer, seu impacto desproporcional sobre as populações mais vulneráveis e a premente necessidade de abordar desigualdades relacionadas ao diagnóstico e tratamento em nível mundial^1^ ^-^ ^2^. 

De acordo com o Instituto Nacional do Câncer (INCA), estima-se a ocorrência de 704 mil novos casos de câncer no Brasil para cada ano do triênio 2023-2025, com destaque para as regiões Sul e Sudeste, que concentram cerca de 70% da incidência^3^ ^-^ ^4^. Excluindo o câncer de pele não-melanoma, espera-se a ocorrência de 483 mil casos novos, sendo 49,5% em homens e 50,5% em mulheres^4^. O tipo de câncer mais incidente em mulheres continua sendo o de mama^4^.

O Brasil possui grande taxa de diagnósticos tardios e de dificuldades no acesso aos serviços para diagnóstico e tratamento do câncer de mama^5^ ^-^ ^6^. Mesmo com avanços políticos e jurídicos para a garantia dos direitos das pacientes oncológicas, a exemplo da aprovação das Lei n° 12.732/2012 (Lei dos 60 dias) e n° 13.896/2019 (Lei dos 30 dias) e pela Política Nacional para a Prevenção e Controle do Câncer, na prática, existem lacunas importantes em termos de acessibilidade, equidade e qualidade na assistência de saúde às mulheres brasileiras com neoplasia maligna de mama^5^ ^,^ ^7^ ^-^ ^9^. 

Para melhorar o acesso ao cuidado de mulheres com câncer de mama da comunidade do Harlem, em Nova Iorque, na década de 1990, a navegação de pacientes surgiu com objetivo de promover o acesso aos sistemas e serviços de saúde em tempo oportuno para a obtenção de melhores desfechos clínicos e mitigar as iniquidades sociais que impactam em tais desfechos^10^. Paulatinamente, a navegação de pacientes se expandiu para outros países, como Canadá e Austrália, e também para assistir a outras condições de saúde^10^. Mais recentemente, essa estratégia de gerenciamento do cuidado chegou à América Latina, e, em 2019, o primeiro programa brasileiro de navegação de pacientes com câncer de mama foi relatado^11^. Em 2023, o Ministério da Saúde, reconhecendo o potencial da estratégia, publicou, junto com a Política Nacional de Prevenção e Controle do Câncer no âmbito do sistema público de saúde, o Programa Nacional de Navegação da Pessoa com Câncer^12^. O objetivo dessa legislação é promover a implementação da navegação de forma efetiva no país^12^.

No Brasil, assim como nos países em que a navegação de pacientes teve origem, o principal profissional que se destaca nesse papel é o enfermeiro, sendo denominado como Enfermeiro Navegador (EN)^13^. Esse profissional mobiliza o seu conhecimento especializado, experiência clínica e competências para promover um cuidado focado nas necessidades dos pacientes, familiares e cuidadores^14^. Assim, o EN supervisiona todo o processo de tratamento, empoderando os pacientes, e atuando como elo entre eles e todo o sistema de saúde, visto que sua atuação almeja identificar barreiras de acesso de diferentes ordens (necessidades de navegação) e superá-las, alcançando resultados mais positivos e em tempo oportuno^13^ ^-^ ^14^.

Há investimentos em pesquisas para aferição do efeito da atuação dos EN em resultados clínicos de mulheres com câncer de mama, no intuito de fortalecer a justificativa da implementação dos programas de navegação nas organizações e redes de saúde^15^ ^-^ ^16^. Estudos indicam que as mulheres navegadas apresentaram níveis significativamente mais baixos de *distress*, ansiedade e depressão durante o tratamento^17^ ^-^ ^18^. O estudo fenomenológico realizado no Peru identificou que o acompanhamento do EN às mulheres com achados anormais em exames de rastreamento para câncer de mama permitiu agilizar as barreiras de acesso, otimizando o diagnóstico definitivo, e, portanto, acelerou o início do tratamento^19^. 

Apesar das evidências internacionais sobre os benefícios da existência dos programas de navegação (PN), ainda é incipiente no Brasil o conhecimento sobre os impactos reais desta estratégia na realidade assistencial, nos serviços e na vida dos pacientes assistidos, incluindo aqueles que vivem com câncer de mama. Tal incipiência pode ser oriunda tanto da recente implementação da navegação no Brasil^11^, como da complexidade do sistema de saúde brasileiro. Além disso, diferenças regionais expressivas que o país apresenta repercutem no acesso e na qualidade do cuidado em saúde. Nesse sentido, o objetivo deste estudo foi comparar os indicadores de tempo diagnóstico-tratamento do cuidado prestado às mulheres com câncer de mama, conforme inclusão em um programa de navegação em enfermagem.

## Método

### Tipo de estudo

Estudo observacional, do tipo coorte retrospectivo, norteado pelo *Strengthening the Reporting of Observational Studies in Epidemiology* (STROBE). Neste tipo de estudo, uma população é acompanhada ao longo do tempo com foco em buscar possíveis associações entre exposição e desfecho e, em se tratando de um estudo retrospectivo, coleta-se a informação pregressa do fator de exposição^20^. 

### Local do estudo

Os campos de inquérito da pesquisa foram o Núcleo de Navegação e o Núcleo de Desfechos, ambos vinculados ao Escritório de Valor (EV) de um hospital de grande porte localizado em Porto Alegre, RS, Brasil. O núcleo de desfechos realiza o monitoramento de pacientes de linhas de cuidado, entre elas a do câncer de mama. Os dados dos pacientes são coletados prospectivamente e acompanhados em tempo real por meio de *dashboards*, incluindo a análise do processo, o perfil clínico do paciente e os desfechos do tratamento ao longo do tempo. O Núcleo de Navegação dessa instituição é o responsável pela implementação de PN nas linhas de cuidado. Atualmente, é composto por uma EN que realiza a navegação na linha de cuidado de câncer de mama, a estruturação e implementação de novos PN, capacitação e treinamento de novos navegadores.

Esse profissional atua na coordenação do cuidado das pacientes, acompanhando-as ao longo de toda a sua jornada de tratamento, desde o momento do seu diagnóstico. Suas atividades contemplam o acolhimento, a escuta ativa e a identificação de barreiras do cuidado que possam vir a comprometer o seu acesso ao tratamento oportuno e seguimento. Cabe ao EN a elaboração e implementação de um plano de cuidados individualizado para cada paciente, orientando paciente e família em relação às consultas, exames e terapias, além do acompanhamento da adesão ao tratamento e o manejo de possíveis sintomas decorrentes das intervenções terapêuticas.

### Período

Entre 01 de outubro de 2022 e 31 de outubro de 2023. 

### População

Pacientes assistidas na Linha de Cuidado de Câncer de Mama, incluídas no banco de dados de monitoramento do Núcleo de Desfechos, acompanhadas ou não pelo PN.

### Critérios de seleção

Critérios de inclusão: pacientes com idade igual ou maior a 18 anos, com diagnóstico de câncer de mama confirmado que não tenha realizado tratamento prévio para a doença, incluídas no banco de dados do núcleo de desfechos até 30 de junho de 2023. Este período-limite de inclusão no banco foi estipulado para garantir que todas as pacientes do estudo tivessem, pelo menos, três meses de acompanhamento de desfechos. 

Critério de exclusão: pacientes que realizaram seu tratamento clínico fora do local do estudo, ainda que tenham registro no banco de dados do local de inquérito; e pacientes com diagnóstico de recidiva da neoplasia no momento de sua inclusão no banco.

### Definição dos participantes

Pacientes assistidas na Linha de Cuidado de Câncer de Mama, incluídas no banco de dados de monitoramento do Núcleo de Desfechos.

O cálculo do tamanho da amostra foi realizado no programa WinPEPI (*Programs for Epidemiologists for Windows*) versão 11.65 e baseado no estudo *Feasibility of Patient Navigation to Improve Breast Cancer Care in Malaysia* ^21^. Considerando um nível de significância de 5%, poder de 90%, um tamanho de efeito da navegação de no mínimo 0,5 desvios-padrão no tempo do diagnóstico até o tratamento e um acréscimo de 20% para eventuais perdas, obteve-se um total mínimo de 204 pacientes, sendo mínimo de 102 em cada grupo.

### Variáveis do estudo

A inclusão no PN foi considerada o fator de comparação. As variáveis independentes categóricas extraídas foram: cor da pele, escolaridade, estado civil, procedência, tipo de convênio (Sistema Único de Saúde ou outros convênios privados) e estágio clínico ao diagnóstico. 

As variáveis quantitativas foram: idade, tempo entre diagnóstico e início de qualquer tratamento, tempo entre diagnóstico e início de tratamento neoadjuvante, tempo entre diagnóstico e tratamento cirúrgico, tempo entre diagnóstico e início da quimioterapia. Os tempos diagnóstico-tratamento foram considerados desfechos (variáveis dependentes), igualmente comparados pelo fator navegação. As medidas de tempos, em dias, foram as seguintes: tempo entre diagnóstico e início de qualquer tratamento, tempo entre diagnóstico e tratamento neoadjuvante e tempo entre diagnóstico e tratamento cirúrgico. 

### Instrumentos utilizados para a coleta das informações

Os dados foram coletados diretamente da plataforma *Research Electronic Data Captur*e (REDCap^®^). 

### Coleta de dados

A coleta de dados foi realizada integralmente em meio eletrônico, com base em sistema informatizado institucional. As variáveis de interesse foram transpostas do sistema institucional, via plataforma REDCap^®^, para planilhas Excel^®^.

As variáveis demográficas e clínicas, incluindo os tempos entre diagnóstico e tratamentos de todas as pacientes, foram coletadas pela equipe do Núcleo de Desfechos, através de formulários específicos e posteriormente lançados no banco de dados institucional via plataforma REDCap^®^. Os formulários foram elaborados pela equipe do setor com base nos critérios da *International Consortium for Health Outcome Measuremen*t (ICHOM) para medidas de desfechos centrados no paciente para câncer de mama não metastático. 

A coleta se dá por meio de revisão de prontuário e contato telefônico com as pacientes nos seguintes momentos: antes do início do tratamento, seis meses e um ano após o tratamento. As pacientes são seguidas anualmente pelo período de até 10 anos.

Os dados referentes ao programa de navegação foram coletados pela enfermeira navegadora na inclusão no programa e no momento de cada um dos atendimentos realizados às pacientes e lançados, também prospectivamente em formulário específico elaborado pela equipe do Núcleo de Navegação no banco de dados institucional, via plataforma REDCap^®^. As pacientes são acompanhadas no PN de acordo com a sua necessidade de navegação, pelo período que necessitarem de suporte por parte do EN, sem limite de tempo, mesmo após finalizarem os tratamentos principais.

### Tratamento e análise dos dados

Os dados foram organizados em planilha Excel^®^ e importados para o *software Statistical Package for the Social Sciences* (SPSS), versão 29.0. Para avaliação da normalidade de distribuição, utilizou-se o teste Kolmogorov-Smirnov. Fez-se análise descritiva e inferencial, em nível de significância de 5%. Para caracterização das participantes, foram descritas medidas de tendência central (mediana e intervalo interquartil) e de frequências absolutas e relativas das variáveis categóricas. Buscou-se parear a amostra considerando a variável idade, a fim de reduzir possíveis vieses de confusão entre os grupos. 

A associação das variáveis sociodemográficas e clínicas com o fator navegação foi verificada pelo teste qui-quadrado de Pearson. Diante da distribuição assimétrica, a comparação das variáveis referentes aos tempos entre diagnóstico e tipos de tratamento das pacientes incluídas ou não no programa de navegação foi verificada pelo teste Mann-Whitney. 

Para atenuar variável potencialmente confundidora, a mesma comparação foi realizada para aferir a diferença de tempos segmentando o grupo de pacientes por modalidade de convênio do atendimento (Sistema Único de Saúde ou Saúde Suplementar), adicionando-se, ainda, o fator navegação. Foram consideradas estatisticamente significantes as diferenças com p≤0,05. 

### Aspectos éticos

A pesquisa seguiu a legislação ética vigente, conforme os termos da Resolução nº 466/2012, do Conselho Nacional de Saúde. Foi submetida ao Comitê de Ética em Pesquisa da instituição do estudo e realizada, após aprovação, sob o parecer nº 6.620.728/2024. A todas as participantes foi fornecido o termo de consentimento livre e esclarecido, enviado de forma eletrônica pelo aplicativo *WhatsApp* ^*®*^, através da plataforma *Google Forms* ^*®*^.

## Resultados

Foram incluídas na linha de cuidado 359 mulheres diagnosticadas com câncer de mama, sem tratamento prévio. A média de tempo de acompanhamento das pacientes incluídas no PN foi de 10 meses.

Da amostra total, 37% (n=133) foram incluídas no PN; 46% (n=165) eram procedentes de Porto Alegre e 64% (n=194) procedentes da grande Porto Alegre e demais municípios do Rio Grande do Sul. 

A média da idade ao diagnóstico de toda a amostra ficou em 58,6±13,6 anos. Quando comparados os grupos de pacientes não navegadas (60,4±13,8 anos) e das navegadas (55,5±12,7 anos), não houve diferença estatística (p=0,232).

Dentre as pacientes navegadas, apresentaram percentuais significativamente maiores as variáveis cor da pele não branca, estadiamento ao diagnóstico III e IV e tipo de tratamento quimioterapia. Na variável tipo de convênio, observou-se que a maioria das pacientes incluídas no PN (93,2%) foi assistida pelo SUS e entre as não incluídas foram as pacientes assistidas pelo sistema de saúde suplementar (63,7%) (Tabela 1).

No que se refere ao intervalo entre o diagnóstico e o início do tratamento neoadjuvante, bem como entre o diagnóstico e o início de qualquer tratamento (Tabela 2), não foram identificadas diferenças estatisticamente significativas entre os grupos, ainda que o tempo tenha sido maior entre as pacientes incluídas no PN em comparação às não incluídas. Por outro lado, quanto ao intervalo entre o diagnóstico e o início do tratamento cirúrgico, observou-se que, nas pacientes navegadas, o tempo foi significativamente superior ao das não navegadas.

Ainda, em relação ao intervalo diagnóstico-tratamento cirúrgico do grupo geral, a mediana de tempo entre diagnóstico e tratamento foi de 94,0 dias, com intervalos interquartílicos variando de -19 a 652 dias. Isso ocorre devido ao fato de que algumas pacientes inicialmente com diagnóstico anatomopatológico benigno podem ter malignidade confirmada apenas após a cirurgia. Nesses casos, o registro do início do tratamento precede a confirmação histopatológica definitiva, gerando intervalos negativos entre diagnóstico e início do tratamento no banco de dados.

**Tabela 1:** Características demográficas e clínicas das pacientes com câncer de mama, de acordo com a inclusão no programa de navegação (n = 359). Porto Alegre, RS, Brasil, 2023

Variável	Total de pacientes	Pacientes incluídas no programa de navegação	Pacientes não incluídas no programa de navegação	p-valor*
	n (%)	n (%)	n (%)	
Convênio				
Sistema Único de Saúde	206 (57,4%)	124 (93,2%)	82 (36,3%)	<0,001
Saúde suplementar	152 (42,6%)	9 (6,8%)	144 (63,7%)	
Cor da pele				
Branca	301 (86%)	101 (77,1%)	200 (91,3%)	<0,001
Não branca	49 (14%)	30 (22,9%)	19 (8,7%)	
Escolaridade				
Ensino fundamental incompleto	84 (24,7%)	19 (35,3%)	42 (19%)	0,001
Ensino fundamental completo ou mais	256 (75,3%)	77 (64,7%)	179 (81%)	
Estado civil				
Casada ou com companheiro	148 (42,4%)	48 (37,8%)	100 (45%)	0,228
Sem companheiro	201 (57,6%)	79 (62,2%)	122 (55%)	
Estadiamento ao diagnóstico				
0, I ou II	227 (79,4%)	77 (64,7%)	150 (89,8%)	<0,001
III ou IV	59 (20,6%)	42 (35,3%)	17 (10,2%)	
Tipo de tratamento				
Cirúrgico	283 (78%)	74 (55,6%)	209 (92,5%)	<0,001
Quimioterapia	150 (41,8%)	98 (73,7%)	52 (23,3%)	<0,001
Hormonioterapia	117 (32,6%)	40 (30,1%)	77 (34,1%)	0,507
Radioterapia	149 (41,5%)	46 (34,6%)	103 (45,6%)	0,054
Quantidade de tratamentos				
Apenas 1	168 (46,9%)	66 (49,6%)	102 (45,3%)	0,499
>1	190 (53,1%)	67 (50,4%)	123 (54,7%)	0,499

*Teste Qui-quadrado de Pearson

**Tabela 2:** Comparação dos tempos entre diagnóstico e modalidades de tratamento entre pacientes com câncer de mama incluídas ou não em programa de navegação (n = 359). Porto Alegre, RS, Brasil, 2023

Variável	Pacientes incluídas no programa de navegação	Pacientes não incluídas no programa	Total de pacientes	p-valor*
	Mediana + Intervalos interquartílicos	Mediana + Intervalos interquartílicos	Mediana + Intervalos interquartílicos	
	(dias)	(dias)	(dias)	
	Mediana	Mediana	Mediana	
Tempo entre diagnóstico e início de qualquer tratamento (P25/75)	75,0 (50,0 / 98,0)	69,0 (42,0 / 112,0)	72,0 (0 / 271,0)	0,394
Tempo entre diagnóstico e tratamento neoadjuvante (P25/75)	72,0 (48,0 / 98,5)	56,0 (45,5 / 99,0)	71,5 (17,0 / 211,0)	0,273
Tempo entre diagnóstico e tratamento cirúrgico (P25/75)	231,0 (79,5 / 284,7)	79,0 (42,0 / 147,2)	94,0 (-19 / 652)	<0,001

*Teste Mann-Whitney

Em relação aos tempos diagnóstico-tratamento no grupo de pacientes atendidas pelo Sistema Único de Saúde (SUS) (Tabela 3), o tempo mediano entre diagnóstico e qualquer tratamento foi de 84 dias. Quando considerado o fator navegação, as pacientes navegadas apresentaram tempos medianos entre diagnóstico e qualquer tratamento (77 dias) e tratamento quimioterápico neoadjuvante (77 dias), significativamente menores do que aquelas não incluídas no PN. No entanto, em relação ao tempo diagnóstico e início de tratamento cirúrgico, as pacientes navegadas apresentaram intervalo significativamente maior do que as não incluídas no PN.

**Tabela 3:** Comparação dos tempos entre diagnóstico e modalidades de tratamento entre pacientes com câncer de mama atendidas pelo Sistema Único de Saúde, incluídas ou não em programa de navegação (n = 153). Porto Alegre, RS, Brasil, 2023

Variável	Pacientes incluídas no programa de navegação	Pacientes não incluídas no programa	Total de pacientes	p-valor*
	Mediana + Intervalos interquartílicos	Mediana + Intervalos interquartílicos	Mediana + Intervalos interquartílicos	
	(dias)	(dias)	(dias)	
	Mediana	Mediana	Mediana	
Tempo entre diagnóstico e início de qualquer tratamento (P25/75)	77,0 (55,0 / 100,0)	100,5 (73,2 / 150,2)	84,0 (60,0 / 122,0)	<0,001
Tempo entre diagnóstico e tratamento neoadjuvante (P25/75)	77,0 (53,0 / 99,7)	85,5 (50,7 / 112,2)	77,0 (52,7 / 101,0)	<0,001
Tempo entre diagnóstico e tratamento cirúrgico (P25/75)	236,0 (85,0 / 285,0)	128,0 (80,0 / 191,5)	152,0 (81,0 / 264,7)	<0,001

No grupo de pacientes atendidas pelo sistema de saúde suplementar (Tabela 4), o tempo mediano entre diagnóstico e qualquer tratamento foi de 42,5 dias. Quando considerada a inclusão ou não no programa de navegação, as pacientes navegadas apresentaram tempos entre diagnóstico e início de qualquer tratamento (38 dias), diagnóstico e início de quimioterapia neoadjuvante (31 dias), significativamente menores que as pacientes não navegadas, que apresentaram intervalos de 47 e 50 dias, respectivamente.

**Tabela 4:** Comparação dos tempos entre diagnóstico e modalidades de tratamento entre pacientes com câncer de mama atendidas pela saúde suplementar, incluídas ou não em programa de navegação (n = 206). Porto Alegre, RS, Brasil, 2023

Variável	Pacientes incluídas no programa de navegação	Pacientes não incluídas no programa	Total de pacientes	p-valor*
	Mediana + Intervalos interquartílicos	Mediana + Intervalos interquartílicos	Mediana + Intervalos interquartílicos	
	(dias)	(dias)	(dias)	
	Mediana	Mediana	Mediana	
Tempo entre diagnóstico e início de qualquer tratamento (P25/75)	38,0 (30,5 / 46,5)	47,0 (28,0 / 62,0)	42,5 (28,2 / 60,5)	<0,001
Tempo entre diagnóstico e tratamento neoadjuvante (P25/75)	31,0 (30,5 / 41,5)	50,0 (17,0 / 56,0)	35,5 (28,5 / 51,5)	<0,001
Tempo entre diagnóstico e tratamento cirúrgico (P25/75)	55,0 (44,0 / 264,0)	49,0 (29,0 / 81,0)	77,0 (52,5 / 101,0)	<0,001

*Teste Mann-Whitney

Em relação aos tempos entre diagnóstico e início de tratamento cirúrgico, a variável apresentou um intervalo significativamente maior nas pacientes navegadas (p<0,05). Quando considerada a inclusão ou não no programa de navegação, segmentando pelo tipo de convênio observa-se que o tempo entre diagnóstico e tratamento cirúrgico persiste maior neste grupo.

## Discussão

Os resultados obtidos neste estudo corroboram o perfil epidemiológico frequentemente descrito na literatura para a população com câncer de mama. A média de idade da amostra foi de 58,6±13,6 anos e não houve diferença entre os grupos. O estudo que analisou o perfil epidemiológico de pessoas com diagnóstico de neoplasia de mama no Brasil evidenciou que a incidência da doença na faixa etária entre 50 a 59 anos é predominante^4^. O mesmo estudo ainda apontou que 98,7% dos casos incidem em mulheres. De acordo com dados do INCA, o câncer de mama feminino é predominante em todas as regiões do Brasil, sendo os casos da doença em homens considerados raros^4^. 

Cor da pele não branca, ensino fundamental incompleto e atendimento pelo SUS foram mais frequentes no grupo incluído no PN. Fatores socioeconômicos como desemprego, baixo nível de escolaridade, pobreza e cor da pele/raça/etnia estão entre os determinantes sociais mais importantes da saúde, inclusive em oncologia^21^ ^-^ ^22^. Aspectos clínicos, características demográficas e demais desigualdades étnicas de acesso aos serviços de saúde têm sido apontadas como influenciadores no acesso ao diagnóstico e tratamento oportuno do câncer de mama nas mulheres^23^ ^-^ ^24^. Assim, no sentido de reduzir a disparidade de acesso, a navegação de pacientes se justifica, ratificada pelos achados deste estudo no que diz respeito aos melhores indicadores de tempo nas modalidades diagnóstico e qualquer tratamento, e diagnóstico e tratamento neoadjuvante entre pacientes tanto atendidas pelo SUS como pela saúde suplementar.

A navegação de pacientes tem como premissa intervir nas disparidades de acesso à atenção em saúde e promover um movimento oportuno dos indivíduos pelo sistema de cuidados^13^ ^-^ ^14^ ^,^ ^24^. Pode-se inferir que o grupo de pacientes navegadas, quando segmentado por modalidade/convênio de atendimento, principalmente nos tempos diagnóstico e início de qualquer tratamento e diagnóstico início de tratamento neoadjuvante, obteve melhor manejo de barreiras para acessar o cuidado, tendo em vista o melhor desempenho entre tais indicadores. A abordagem realizada pelo navegador proporciona aos pacientes apoio e comunicação constante, fornecendo informações fundamentais sobre o diagnóstico e jornada terapêutica e ajudando a manter uma melhor adesão ao tratamento^25^. Logo, este estudo vai ao encontro das premissas da navegação de pacientes.

Em relação ao estadiamento no diagnóstico, 74,5% da amostra apresentaram neoplasia nos estágios 0, I e II, sem diferença entre os grupos. Entre as pacientes incluídas no programa de navegação, evidenciou-se um volume maior de diagnósticos com estágio III e IV (35,3%) em relação às pacientes não incluídas (10,2%). Isto pode ser explicado pelo fato de que as pacientes atendidas pelo SUS correspondiam majoritariamente ao grupo “navegadas”. Em outras palavras, apesar de a navegação ter melhorado o tempo de acesso ao tratamento neoadjuvante e a qualquer tratamento, ainda assim, as pacientes do sistema público demoraram muito mais para ter acesso aos tratamentos do que aquelas originárias do sistema suplementar, o que pode ter repercutido no estadiamento mais avançado do câncer. Apesar de essa realidade corroborar as iniquidades sociais do Brasil, as pacientes do SUS que foram incluídas no PN tiveram resultados de tempo de acesso ao tratamento clínico significativamente melhores do que aquelas não incluídas. Isso sugere a necessidade de investimento governamental nessa modalidade de atuação do enfermeiro, em especial, em doenças progressivas.

O estudo transversal, analítico e retrospectivo realizado em 2024 em um hospital público de Brasília, identificou que 35% das pacientes foram diagnosticadas com estadiamento III, demonstrando o diagnóstico mais tardio nessas mulheres^26^. Outro estudo, realizado no Rio de Janeiro, identificou a prevalência de 43,7% dos diagnósticos em estágio avançado (estágios III e IV)^25^. Essa condição foi observada entre mulheres com idade média de 49 anos, cor de pele não branca, que viviam sem companheiro e encaminhadas através do SUS^27^. Embora em menor proporção do que os estudos citados, nossos achados demonstram que há espaço de melhoria na redução de diagnósticos em estágios III e IV, independente da inclusão ou não no programa de navegação de pacientes. 

O manejo do câncer de mama requer uma abordagem personalizada, levando em conta, entre outros, o estadiamento da doença, os resultados da imunohistoquímica e as dimensões do tumor^28^. Atualmente, a intervenção cirúrgica para a retirada do tumor não é mais a única etapa crítica do tratamento, mas sim uma parte de um processo amplo enfrentado pelas mulheres^28^ ^-^ ^29^. Quimioterapia, radioterapia, hormonioterapia e imunoterapia, por exemplo, são formas complementares de tratamento que, ao longo do tempo, têm contribuído significativamente para aumentar as taxas de sobrevivência e o período livre de avanço da doença^29^. Considerando o número de modalidades de tratamento realizadas, na totalidade da amostra, 53,1% das pacientes realizaram mais de um tratamento, não havendo diferença significativa entre as pacientes navegadas e não navegadas. O dado sugere que há disponibilidade de diversas modalidades de tratamento no serviço pesquisado, independente do suporte ou não fornecido pelo EN.

Em relação aos tipos de tratamento realizado, predominou o tratamento quimioterápico (73,7%) entre as pacientes navegadas. As pacientes em quimioterapia podem apresentar diversos efeitos adversos e permanecem em tratamento vários meses, dependendo da terapia indicada, o que pode dificultar o seguimento ou mesmo a desistência do tratamento^30^ ^-^ ^32^. Os sinais e sintomas do tratamento quimioterápico como náuseas, vômitos, fadiga, mucosite, neutropenia febril são alguns efeitos colaterais que acabam por contribuir com isso^30^ ^-^ ^32^. A adesão ao tratamento, nesse sentido, é um processo multidimensional que recebe a inferência de aspectos sociais, econômicos e individuais da pessoa e requer ajuda de profissionais da saúde capacitados para o processo educativo em saúde^30^ ^-^ ^32^. Dessa forma, a coordenação do cuidado realizada pelo EN é focada em garantir melhor aderência ao tratamento e reduzir taxas de abandono devido a esses efeitos colaterais.

Entre as pacientes não navegadas, houve o predomínio de mulheres com realização de procedimentos cirúrgicos (92,5%). Isso pode ser atribuído ao fato de que as pacientes que realizam somente esta modalidade de tratamento não seguem o tratamento clínico no serviço, por isso não são incluídas no PN, e, um dos critérios de inclusão no PN é realizar todo o tratamento no serviço. Outra hipótese para este achado é a maior concentração de pacientes não navegadas no grupo de saúde suplementar, o qual apresentou menor tempo de acesso ao tratamento cirúrgico, e, consequentemente, podem não ter necessitado de outra modalidade de assistência além da cirurgia. 

No Brasil, foi estabelecido pelo Ministério da Saúde (MS), por meio da Lei nº 12.732/2012, um prazo de 60 dias para o início do tratamento de pacientes com diagnóstico de neoplasia maligna, contado a partir da confirmação diagnóstica por laudo de exame anatomopatológico até o primeiro dia de realização da modalidade terapêutica indicada^8^. Pesquisas apontam que esse intervalo, quando superior a 60 dias, pode estar associado a pior prognóstico^33^ ^-^ ^34^. Estudo realizado nos Estados Unidos avaliou o tempo entre diagnóstico e tratamento de mulheres de baixa renda com câncer de mama e apontou que cerca de 10% esperam um intervalo maior ou igual a 60 dias para iniciar o tratamento^33^. Esse fator está associado a uma diminuição da sobrevida global em 5 anos de 66% neste grupo^33^. 

Os resultados do presente estudo evidenciam que apesar de próximo à recomendação legal^8^, o tempo entre diagnóstico e qualquer tratamento e diagnóstico e início de tratamento neoadjuvante no local da pesquisa ainda demanda ações para melhoria deste intervalo, principalmente nas pacientes do sistema público de saúde. Essa é uma realidade que reafirma que o SUS demanda investimento significativo e permanente, bem como, reforça um olhar crítico para a dimensão de sua premissa de integralidade, incluindo-se aí a otimização do tempo de acesso. Conforme atestam os resultados ora descritos, a atuação do EN pode ser uma estratégia promissora para tal fim. 

Na análise da variável tempo entre diagnóstico e início de tratamento cirúrgico, ficou evidenciado que principalmente as pacientes atendidas pelo SUS apresentaram um intervalo estendido para a sua realização. Tal achado pode estar associado ao fato de que, na análise desta variável, não foram considerados somente os procedimentos cirúrgicos como primeiro tratamento, estando incluídos nesse resultado os procedimentos realizados após finalização do tratamento sistêmico neoadjuvante (quimioterapia ou hormonioterapia). Isso é explicado, pois, no momento da inclusão da data de realização da cirurgia no banco de dados, não é especificado se isso foi anterior ou posterior ao tratamento sistêmico neoadjuvante, inviabilizando essa seleção para a realização de uma análise mais acurada. 

A neoadjuvância refere-se à realização de um tratamento sistêmico antes do tratamento considerado de maior impacto curativo, usualmente a cirurgia^35^ ^-^ ^36^ e tem duração média, no câncer de mama, de 4 a 6 meses, dependendo do protocolo terapêutico indicado^37^. Anteriormente, estava reservada para pacientes com tumores localmente avançados ou inoperáveis e tinha o objetivo de somente reduzir o tamanho do tumor para permitir uma cirurgia menos extensa e mutiladora^35^ ^-^ ^36^. No entanto, atualmente, mais pacientes com câncer de mama em estágio inicial têm indicação deste tipo de tratamento^35^ ^-^ ^36^. Pacientes com estadiamento inicial podem ser candidatas à indicação de neoadjuvância por potencial benefício em variáveis preditivas e prognósticas. Ainda, esse tipo de tratamento tem proporcionado a oportunidade de realização de cirurgias mais conservadoras, a fim de reduzir a necessidade de mastectomias totais^35^. Dessa forma, o intervalo estendido até o tratamento cirúrgico pode estar ligado ao volume de cirurgias pós-neoadjuvância, considerando que 53% dos pacientes fazem mais de um tratamento e o estadiamento ao diagnóstico, uma vez que um percentual significativamente maior de pacientes da amostra (89,8%), apresentavam estágios iniciais ao diagnóstico. 

Ao analisar o grupo geral de pacientes, sem segmentação por convênio, observou-se que não houve diferença significativa entre os indicadores de tempo de acesso mensurados. As pacientes navegadas apresentaram um tempo maior de acesso aos tratamentos. Todavia, importa destacar que a variável tipo de convênio influenciou neste achado, tendo em vista que os indicadores de tempo diagnóstico-tratamento das mulheres atendidas na saúde suplementar são muito menores do que aquelas atendidas no SUS, independentes se assistidas ou não pelo enfermeiro navegador. Isso ratifica que as iniquidades sociais são um marcador no acesso ao cuidado do câncer de mama^4^ ^-^ ^6^; e, tendo em vista os resultados segmentados por tipo de convênio, a navegação em enfermagem pode constituir uma estratégia profícua de enfrentamento dessas iniquidades. 

Os achados relacionados aos tempos diagnóstico-tratamento reforçam a preocupante disparidade principalmente entre as pacientes atendidas pelo SUS, uma vez que essa parcela da amostra contribuiu para o aumento no tempo de acesso ao cuidado. Diante disso, é fundamental garantir que as pacientes do sistema público de saúde tenham acesso a programas de rastreio e diagnóstico precoce do câncer, possibilitando um contato mais ágil com o EN, o que poderá impactar positivamente no tempo de acesso ao cuidado.

Um estudo brasileiro, realizado com base na análise dos dados coletados no DATASUS e PAINEL-Oncologia, evidenciou que 43,6% dos indivíduos diagnosticados com neoplasia de mama pelo SUS apresentaram intervalos entre diagnóstico e tratamento acima 60 dias. Ainda, somente 21,2% iniciaram tratamento entre 31 e 60 dias^37^. A pesquisa transversal realizada no Piauí apontou que, entre os anos de 2016 e 2017, 71% das mulheres diagnosticadas com câncer de mama apresentaram em um intervalo mediano de 83,8 dias para iniciar o tratamento oncológico indicado^38^. Os autores atribuíram tal atraso, principalmente, a uma desarticulação da rede de atenção em oncologia e sugeriram que há necessidade de coordenação dos fluxos e organização dos pontos de atenção especializada^38^. O enfermeiro desponta neste processo, visto que, por natureza, promove a gestão do cuidado, o que pode contribuir para a articulação da assistência nos pontos de atenção da rede de saúde. 

A coorte retrospectiva realizada em Minas Gerais evidenciou o tempo mediano de 71,5 dias entre diagnóstico e tratamento em mulheres tratadas no sistema público e apontou que fatores relacionados às desigualdades sociais podem influenciar no acesso aos cuidados no câncer de mama^39^. Os achados desta pesquisa demonstram consonância com o que é apontado pela literatura. 

Em relação às pacientes do sistema suplementar, também ficou evidenciado que as pacientes navegadas apresentaram medianas de tempo diagnóstico-qualquer tratamento e diagnóstico-tratamento neoadjuvante significativamente menores do que as pacientes não navegadas. Isso é interessante e demonstra que a navegação é profícua independente do convênio de atendimento. No caso da saúde suplementar, além de melhorar desfechos clínicos, o EN pode contribuir para a rentabilidade e sustentabilidade financeira dos serviços, uma vez que o tempo oportuno é fator de implicação neste aspecto. 

O relato de experiência descreveu a atuação de EN na atenção a pacientes com câncer de mama em um hospital privado de um município no sul do país e demonstrou que, através de ações realizadas pelas profissionais, foi possível garantir que as pacientes incluídas no PN do serviço recebessem seu tratamento em tempo hábil^40^. Além disso, a revisão sistemática liderada por pesquisadores coreanos indicou que os estudos (n=6) incluídos evidenciaram que os enfermeiros navegadores reduziram o número de dias de intervalo entre diagnóstico e tratamento em cerca de 17,5 dias^41^. Tais evidências agregam contundência aos achados deste estudo, confirmando que o EN, através da coordenação do cuidado, mitigação de barreiras de acesso e orientações sobre o diagnóstico e jornada de tratamento é capaz de melhorar o acesso em saúde, inclusive em situações onde o tempo é tão importante como o câncer. 

Outro estudo publicado recentemente demonstrou que, após a implementação de um programa de navegação para pacientes com câncer de mama no sul do país, foi possível reduzir o tempo entre diagnóstico e início de tratamento neoadjuvante em cerca de 26%^42^. Os intervalos de tempos (diagnóstico-tratamento neoadjuvante e diagnóstico-qualquer tratamento) significativamente mais curtos apresentados pelas pacientes incluídas no PN em relação às não incluídas chegou a uma diferença de 23 dias naquelas tratadas no sistema público e 29 dias no sistema privado. Atrasos para o início do tratamento oncológico são conhecidos por comprometer seriamente os desfechos clínicos. Retardos prolongados no intervalo entre diagnóstico-tratamento, estão diretamente associados à menor sobrevida das pacientes com câncer de mama^43^, justificando que esforços precisam ser feitos para mitigar e resolver barreiras que possam interferir nesse indicador. Isso demonstra que os achados corroboram com o que têm sido evidenciado em publicações científicas, comprovando que, através da navegação em enfermagem, é possível impactar de forma positiva nos tempos considerados críticos para a obtenção dos melhores desfechos clínicos. 

É prudente assumir como limitações deste estudo a ausência de manejo de outras variáveis potencialmente confundidoras além da modalidade de convênio no atendimento, e a limitação natural de possível inconsistência de registros. Análises mais robustas considerando outros desfechos além do tempo de acesso ao tratamento são altamente recomendadas. No entanto, o estudo é relevante e confere legitimidade científica para sustentar a difusão da navegação de pacientes coordenada por enfermeiros. 

Este estudo traz relevante contribuição para o avanço do conhecimento ao demonstrar, por meio da comparação dos tempos diagnóstico-tratamento, a potencial efetividade de um programa de navegação para pacientes com câncer de mama. Assim, esta pesquisa destaca a importância da navegação como uma estratégia capaz de otimizar o acesso à assistência oncológica, minimizar barreiras do cuidado no sistema de saúde e potencialmente melhorar os desfechos clínicos. Os dados apresentados podem, potencialmente, apoiar a implementação de políticas de saúde focadas em reduzir atrasos no tratamento oncológico.

## Conclusão

Pacientes com câncer de mama incluídas em um programa de navegação liderado por enfermeiro tiveram indicadores de tempo de acesso ao tratamento em geral e tratamento quimioterápico neoadjuvante significativamente menores do que as pacientes não incluídas. Isso foi evidenciado principalmente quando os grupos de pacientes navegadas e não navegadas foram segmentados pelo tipo de convênio (público ou privado) no atendimento. Em ambos os casos, as pacientes navegadas tiveram tempos significativamente melhores, embora as pacientes oriundas do sistema público ainda demorem expressivamente mais para ter acesso ao cuidado. 

Estratégias desenvolvidas pelo EN no PN, tais como avaliação das barreiras de acesso à assistência, elaboração e implementação de um plano de cuidados individualizado e a realização de ações de educação em saúde direcionadas a pacientes e familiares em relação ao diagnóstico e à jornada de tratamento, podem contribuir de forma positiva nestes intervalos críticos. Logo, além de evidenciar a efetividade no trabalho do Enfermeiro Navegador, este estudo atesta a necessidade de investimento na implementação da navegação nos serviços de saúde e em programas de rastreamento e diagnóstico precoce do câncer de mama no SUS. É importante salientar que futuras pesquisas, especialmente com a realização de estudos prospectivos e estudos controlados, precisam ser realizadas para avaliar desfechos mais amplos, como sobrevida, qualidade de adesão terapêutica e qualidade de vida, a fim de fortalecer a evidência sobre a efetividade da navegação em enfermagem no cuidado oncológico.

## Data Availability

Todos os dados gerados ou analisados durante este estudo estão incluídos neste artigo publicado.

# Translation: Programa de navegación: comparación de los tiempos diagnóstico-tratamiento entre pacientes con cáncer de mama*

**DOI:** 10.1590/1518-8345.8049.4845

**Authors:** Fernanda Felipe Pautasso, Maiara Anchau Floriani, Nicole de Moraes Pertile, João Lucas Campos de Oliveira

**Affiliations:** 1 Universidade Federal do Rio Grande do Sul, Escola de Enfermagem, Porto Alegre, RS, Brasil.; 2 Santa Casa de Porto Alegre, Escritório de Valor, Porto Alegre, RS, Brasil.; 3 Santa Casa de Porto Alegre, Escritório da Qualidade e Valor, Porto Alegre, RS, Brasil.

**Keywords:** Enfermería, Navegación del Paciente, Oncología Médica, Cáncer de Mama, Tiempo de Tratamiento, Rol de la Enfermera

## Abstract

**Objetivo:**

comparar los indicadores de tiempo diagnóstico-tratamiento del cuidado prestado a mujeres con cáncer de mama, según la inclusión en un programa de navegación en enfermería.

**Método:**

cohorte retrospectiva, realizada en un hospital de gran porte. Se incluyeron pacientes con cáncer de mama, navegadas (n=133) o no (n=226). Se analizaron variables sociodemográficas y clínicas; y los tiempos (en días) de diagnóstico e inicio de diferentes modalidades de tratamiento constituyeron las variables dependientes. Se realizó análisis descriptivo e inferencial, con nivel de significancia de 5%.

**Resultados:**

pacientes navegadas atendidas por el Sistema Único de Salud presentaron tiempos medianos entre diagnóstico y cualquier tratamiento (77,0) y quimioterapia neoadyuvante (77,0) significativamente menores (p-valor<0,001) que las no incluidas (100,5 y 112,2, respectivamente). Pacientes navegadas por el sistema de salud suplementaria también presentaron menor tiempo de acceso tanto al tratamiento en general (38,0 *versus* 47,0; p-valor<0,001) como al quimioterápico neoadyuvante (31,0 *versus* 50,0; p-valor<0,001). Sin considerar el convenio de atención, no se verificaron diferencias significativas.

**Conclusión:**

el programa de navegación impactó positivamente en la accesibilidad diagnóstico-tratamiento del cáncer de mama. Se recomienda invertir en esta modalidad de gestión del cuidado promovida por el enfermero, con mayor énfasis en el Sistema Único de Salud.

## Destacados

**(1)** Se evidenció reducción del tiempo diagnóstico-tratamiento en las pacientes navegadas. **(2)** Se evidenció la efectividad del trabajo del enfermero navegador en el cuidado. **(3)** Se señala la necesidad de invertir en la implementación de programas de navegación.

## Introducción

A comienzos de 2024, la Agencia Internacional para la Investigación del Cáncer (IARC) de la Organización Mundial de la Salud (OMS) divulgó estimaciones preocupantes relacionadas con la carga mundial de la enfermedad^1^. Entre las estadísticas, se destacó el creciente peso del cáncer, su impacto desproporcionado sobre las poblaciones más vulnerables y la apremiante necesidad de abordar desigualdades relacionadas con el diagnóstico y el tratamiento a nivel mundial^1^ ^-^ ^2^.

De acuerdo con el *Instituto Nacional de Câncer* (INCA), se estima la ocurrencia de 704 mil nuevos casos de cáncer en Brasil para cada año del trienio 2023-2025, con destaque para las regiones Sur y Sudeste, que concentran cerca de 70% de la incidencia^3^ ^-^ ^4^. Excluyendo el cáncer de piel no melanoma, se espera la ocurrencia de 483 mil casos nuevos, de los cuales 49,5% en hombres y 50,5% en mujeres^4^. El tipo de cáncer más incidente en mujeres continúa siendo el de mama^4^.

Brasil presenta una elevada tasa de diagnósticos tardíos y dificultades en el acceso a los servicios para diagnóstico y tratamiento del cáncer de mama^5^ ^-^ ^6^. Incluso con avances políticos y jurídicos para la garantía de los derechos de las pacientes oncológicas, como la aprobación de las Leyes n.º 12.732/2012 (Ley de los 60 días) y n.º 13.896/2019 (Ley de los 30 días) y de la Política Nacional para la Prevención y el Control del Cáncer, en la práctica existen lagunas importantes en términos de accesibilidad, equidad y calidad en la atención de salud a las mujeres brasileñas con neoplasia maligna de mama^5^ ^,^ ^7^ ^-^ ^9^.

Para mejorar el acceso al cuidado de mujeres con cáncer de mama de la comunidad de Harlem, en Nueva York, en la década de 1990, la navegación de pacientes surgió con el objetivo de promover el acceso a los sistemas y servicios de salud en tiempo oportuno para la obtención de mejores desenlaces clínicos y mitigar las inequidades sociales que impactan dichos desenlaces^10^. Paulatinamente, la navegación de pacientes se expandió a otros países, como Canadá y Australia, y también para otras condiciones de salud^10^. Más recientemente, esta estrategia de gestión del cuidado llegó a América Latina y, en 2019, se informó el primer programa brasileño de navegación de pacientes con cáncer de mama^11^. En 2023, el Ministerio de Salud, reconociendo el potencial de la estrategia, publicó, junto con la Política Nacional de Prevención y Control del Cáncer en el ámbito del sistema público de salud, el Programa Nacional de Navegación de la Persona con Cáncer^12^. El objetivo de esta legislación es promover la implementación efectiva de la navegación en el país^12^.

En Brasil, así como en los países donde se originó la navegación de pacientes, el principal profesional que se destaca en este rol es el enfermero, denominado Enfermero Navegador (EN)^13^. Este profesional moviliza su conocimiento especializado, experiencia clínica y competencias para promover un cuidado centrado en las necesidades de los pacientes, familiares y cuidadores^14^. Así, el EN supervisa todo el proceso de tratamiento, empoderando a los pacientes y actuando como enlace entre ellos y todo el sistema de salud, ya que su actuación tiene por objetivo identificar barreras de acceso de diferentes órdenes (necesidades de navegación) y superarlas, alcanzando resultados más positivos y en tiempo oportuno^13^ ^-^ ^14^.

Se están realizando inversiones en investigaciones para medir el efecto de la actuación de los EN en los resultados clínicos de mujeres con cáncer de mama, con el fin de fortalecer la justificación para la implementación de programas de navegación en las organizaciones y redes de salud^15^ ^-^ ^16^. Estudios indican que las mujeres navegadas presentaron niveles significativamente más bajos de *distress*, ansiedad y depresión durante el tratamiento^17^ ^-^ ^18^. El estudio fenomenológico realizado en Perú identificó que el acompañamiento del EN a mujeres con hallazgos anormales en exámenes de tamizaje para cáncer de mama permitió superar con mayor rapidez las barreras de acceso, optimizando el diagnóstico definitivo y, por lo tanto, acelerando el inicio del tratamiento^19^.

A pesar de las evidencias internacionales sobre los beneficios de la existencia de programas de navegación (PN), en Brasil todavía es incipiente el conocimiento sobre los impactos reales de esta estrategia en la realidad asistencial, en los servicios y en la vida de los pacientes atendidos, incluyendo aquellos que viven con cáncer de mama. Tal incipiencia puede deberse tanto a la reciente implementación de la navegación en Brasil^11^, como a la complejidad del sistema de salud brasileño. Además, las marcadas diferencias regionales que presenta el país repercuten en el acceso y en la calidad del cuidado en salud. En este sentido, el objetivo de este estudio fue comparar los indicadores de tiempo diagnóstico-tratamiento del cuidado prestado a mujeres con cáncer de mama, según la inclusión en un programa de navegación en enfermería.

## Método

### Tipo de estudio

Estudio observacional, del tipo cohorte retrospectiva, guiado por el *Strengthening the Reporting of Observational Studies in Epidemiology* (STROBE). En este tipo de estudio, una población es acompañada a lo largo del tiempo con el foco puesto en buscar posibles asociaciones entre exposición y desenlace y, tratándose de un estudio retrospectivo, se recoge la información pasada del factor de exposición^20^. 

### Lugar del estudio

Los escenarios del estudio fueron el Núcleo de Navegación y el Núcleo de Resultados, ambos vinculados al *Escritório de Valor* (EV) de un hospital de gran porte ubicado en Porto Alegre, RS, Brasil. El Núcleo de Resultados realiza el monitoreo de pacientes de líneas de cuidado, entre ellas la de cáncer de mama. Los datos de las pacientes se recopilan prospectivamente y se acompañan en tiempo real por medio de *dashboards*, incluyendo el análisis del proceso, el perfil clínico de la paciente y los resultados del tratamiento a lo largo del tiempo. El Núcleo de Navegación de esa institución es el responsable de la implementación de PN en las líneas de cuidado. Actualmente, está compuesto por una EN que realiza la navegación en la Línea de Cuidado de Cáncer de Mama, la estructuración e implementación de nuevos PN, así como la capacitación y el entrenamiento de nuevos navegadores.

Este profesional coordina el cuidado de las pacientes, acompañándolas a lo largo de toda su trayectoria de tratamiento, desde el momento del diagnóstico. Sus actividades contemplan la acogida, la escucha activa y la identificación de barreras en el cuidado que puedan llegar a comprometer su acceso al tratamiento oportuno y al seguimiento. Corresponde al EN la elaboración e implementación de un plan de cuidados individualizado para cada paciente, orientando a la paciente y a la familia en relación con las consultas, exámenes y terapias, además del acompañamiento de la adherencia al tratamiento y del manejo de posibles síntomas derivados de las intervenciones terapéuticas.

### Período

Entre el 1 de octubre de 2022 y el 31 de octubre de 2023.

### Población

Pacientes atendidas en la Línea de Cuidado de Cáncer de Mama, incluidas en la base de datos de monitoreo del Núcleo de Resultados, acompañadas o no por el PN.

### Criterios de selección

Criterios de inclusión: pacientes con edad igual o superior a 18 años, con diagnóstico de cáncer de mama confirmado que no hubieran realizado tratamiento previo para la enfermedad, incluidas en la base de datos del Núcleo de Resultados hasta el 30 de junio de 2023. Este período límite de inclusión en la base se estipuló para garantizar que todas las pacientes del estudio tuvieran, por lo menos, tres meses de acompañamiento de resultados.

Criterio de exclusión: pacientes que realizaron su tratamiento clínico fuera del lugar del estudio, aunque tuvieran registro en la base de datos del lugar de investigación; y pacientes con diagnóstico de recidiva de la neoplasia en el momento de su inclusión en la base.

### Definición de las participantes

Pacientes atendidas en la Línea de Cuidado de Cáncer de Mama, incluidas en la base de datos de monitoreo del Núcleo de Resultados.

El cálculo del tamaño de la muestra se realizó en el programa WinPEPI (*Programs for Epidemiologists for Windows*) versión 11.65 y se basó en el estudio *Feasibility of Patient Navigation to Improve Breast Cancer Care in Malaysia* ^21^. Considerando un nivel de significancia de 5%, poder de 90%, un tamaño de efecto de la navegación de por lo menos 0,5 desviaciones estándar en el tiempo desde el diagnóstico hasta el tratamiento y un incremento del 20% para posibles pérdidas, se obtuvo un total mínimo de 204 pacientes, siendo un mínimo de 102 en cada grupo.

### Variables del estudio

La inclusión en el PN se consideró el factor de comparación. Las variables independientes categóricas extraídas fueron: color de piel, escolaridad, estado civil, procedencia, tipo de convenio (Sistema Único de Salud u otros convenios privados) y estadio clínico al diagnóstico.

Las variables cuantitativas fueron: edad, tiempo entre diagnóstico e inicio de cualquier tratamiento, tiempo entre diagnóstico e inicio de tratamiento neoadyuvante, tiempo entre diagnóstico y tratamiento quirúrgico, tiempo entre diagnóstico e inicio de la quimioterapia. Los tiempos diagnóstico-tratamiento se consideraron desenlaces (variables dependientes), igualmente comparados según el factor navegación. Las medidas de tiempo, en días, fueron las siguientes: tiempo entre diagnóstico e inicio de cualquier tratamiento, tiempo entre diagnóstico y tratamiento neoadyuvante y tiempo entre diagnóstico y tratamiento quirúrgico. 

### Instrumentos utilizados para la recogida de la información

Los datos se recopilaron directamente de la plataforma *Research Electronic Data Captur*e (REDCap^®^). 

### Recogida de datos

La recogida de datos se realizó íntegramente en formato electrónico, con base en un sistema informatizado institucional. Las variables de interés se transfirieron del sistema institucional, por medio de la plataforma (REDCap^®^), a hojas de cálculo Excel^®^.

Las variables demográficas y clínicas, incluidos los tiempos entre diagnóstico y tratamientos de todas las pacientes, fueron recogidas por el equipo del Núcleo de Resultados mediante formularios específicos y posteriormente cargadas en la base de datos institucional a través de la plataforma REDCap^®^. Los formularios fueron elaborados por el equipo del sector con base en los criterios del *International Consortium for Health Outcome Measurement* (ICHOM) para medidas de desenlaces centrados en el paciente para cáncer de mama no metastásico.

La recogida se realiza por medio de revisión de historia clínica y contacto telefónico con las pacientes en los siguientes momentos: antes del inicio del tratamiento, seis meses y un año después del tratamiento. Las pacientes son seguidas anualmente por un período de hasta 10 años.

Los datos referentes al programa de navegación fueron recogidos por la enfermera navegadora en la inclusión en el programa y en el momento de cada una de las atenciones realizadas a las pacientes y cargados, también de forma prospectiva, en un formulario específico elaborado por el equipo del Núcleo de Navegación en la base de datos institucional, por medio de la plataforma REDCap^®^. Las pacientes son acompañadas en el PN de acuerdo con su necesidad de navegación, durante el período en que requieran apoyo por parte del EN, sin límite de tiempo, incluso después de finalizar los tratamientos principales.

### Tratamiento y análisis de los datos

Los datos se organizaron en una hoja de cálculo Excel^®^ y se importaron al *software Statistical Package for the Social Sciences* (SPSS), versión 29.0. Para la evaluación de la normalidad de la distribución se utilizó la prueba Kolmogorov-Smirnov. Se realizó análisis descriptivo e inferencial, con nivel de significancia de 5%. Para la caracterización de las participantes, se describieron medidas de tendencia central (mediana e intervalo intercuartílico) y frecuencias absolutas y relativas de las variables categóricas. Se procuró emparejar la muestra considerando la variable edad, con el fin de reducir posibles sesgos de confusión entre los grupos.

La asociación de las variables sociodemográficas y clínicas con el factor navegación se verificó mediante la prueba de chi-cuadrado de Pearson. Dada la distribución asimétrica, la comparación de las variables referentes a los tiempos entre diagnóstico y tipos de tratamiento de las pacientes incluidas o no en el programa de navegación se verificó mediante la prueba de Mann-Whitney.

Para atenuar una variable potencialmente confusora, se realizó la misma comparación para evaluar la diferencia de tiempos segmentando el grupo de pacientes por modalidad de convenio de atención (Sistema Único de Salud o salud suplementaria), añadiéndose, además, el factor navegación. Se consideraron estadísticamente significativas las diferencias con p≤0,05.

### Aspectos éticos

La investigación siguió la legislación ética vigente, conforme a los términos de la Resolución n.º 466/2012, del *Conselho Nacional de Saúde*. Fue sometida al Comité de Ética en Investigación de la institución del estudio y realizada, tras su aprobación, bajo el dictamen n.º 6.620.728/2024. A todas las participantes se les proporcionó el término de consentimiento libre e informado, enviado de forma electrónica por la aplicación WhatsApp^®^, por medio de la plataforma Google *Forms* ^*®*^.

## Resultados

Se incluyeron en la línea de cuidado 359 mujeres diagnosticadas con cáncer de mama, sin tratamiento previo. La media del tiempo de seguimiento de las pacientes incluidas en el PN fue de 10 meses.

De la muestra total, 37% (n=133) fueron incluidas en el PN; 46% (n=165) eran procedentes de Porto Alegre y 64% (n=194) procedentes del Gran Porto Alegre y demás municipios de Rio Grande do Sul.

La media de edad al diagnóstico de toda la muestra fue de 58,6±13,6 años. Al comparar los grupos de pacientes no navegadas (60,4±13,8 años) y navegadas (55,5±12,7 años), no hubo diferencia estadística (p=0,232).

Entre las pacientes navegadas, presentaron porcentajes significativamente mayores las variables color de piel no blanca, estadificación al diagnóstico III y IV y tipo de tratamiento quimioterapia. En la variable tipo de convenio, se observó que la mayoría de las pacientes incluidas en el PN (93,2%) fueron asistidas por el SUS y, entre las no incluidas, fueron mayoritarias las pacientes asistidas por el sistema de salud suplementaria (63,7%) (Tabla 1).

En lo que se refiere al intervalo entre el diagnóstico y el inicio del tratamiento neoadyuvante, así como entre el diagnóstico y el inicio de cualquier tratamiento (Tabla 2), no se identificaron diferencias estadísticamente significativas entre los grupos, aunque el tiempo fue mayor entre las pacientes incluidas en el PN en comparación con las no incluidas. Por otro lado, en cuanto al intervalo entre el diagnóstico y el inicio del tratamiento quirúrgico, se observó que, en las pacientes navegadas, el tiempo fue significativamente superior al de las no navegadas.

Asimismo, en relación con el intervalo diagnóstico-tratamiento quirúrgico del grupo general, la mediana del tiempo entre diagnóstico y tratamiento fue de 94,0 días, con intervalos intercuartílicos que variaron de -19 a 652 días. Esto ocurre debido a que algunas pacientes inicialmente con diagnóstico anatomopatológico benigno pueden tener la malignidad confirmada solo después de la cirugía. En estos casos, el registro del inicio del tratamiento precede a la confirmación histopatológica definitiva, generando intervalos negativos entre el diagnóstico y el inicio del tratamiento en la base de datos.

**Tabla 1:** Características demográficas y clínicas de las pacientes con cáncer de mama, de acuerdo con la inclusión en el programa de navegación (n = 359). Porto Alegre, RS, Brasil, 2023

Variable	Total de pacientes n (%)	Pacientes incluidas en el programa de navegación n (%)	Pacientes no incluidas en el programa de navegación n (%)	p-valor*
Convenio				
Sistema Único de Salud	206 (57,4%)	124 (93,2%)	82 (36,3%)	<0,001
Salud suplementaria	152 (42,6%)	9 (6,8%)	144 (63,7%)	
Color de piel				
Blanca	301 (86%)	101 (77,1%)	200 (91,3%)	<0,001
No blanca	49 (14%)	30 (22,9%)	19 (8,7%)	
Escolaridad				
Educación primaria incompleta	84 (24,7%)	19 (35,3%)	42 (19%)	0,001
Educación primaria completa o más	256 (75,3%)	77 (64,7%)	179 (81%)	
Estado civil				
Casada o con pareja	148 (42,4%)	48 (37,8%)	100 (45%)	0,228
Sin pareja	201 (57,6%)	79 (62,2%)	122 (55%)	
Estadificación al diagnóstico				
0, I o II	227 (79,4%)	77 (64,7%)	150 (89,8%)	<0,001
III u IV	59 (20,6%)	42 (35,3%)	17 (10,2%)	
Tipo de tratamiento				
Quirúrgico	283 (78%)	74 (55,6%)	209 (92,5%)	<0,001
Quimioterapia	150 (41,8%)	98 (73,7%)	52 (23,3%)	<0,001
Hormonoterapia	117 (32,6%)	40 (30,1%)	77 (34,1%)	0,507
Radioterapia	149 (41,5%)	46 (34,6%)	103 (45,6%)	0,054
Cantidad de tratamientos				
Solo 1	168 (46,9%)	66 (49,6%)	102 (45,3%)	0,499
>1	190 (53,1%)	67 (50,4%)	123 (54,7%)	0,499

*Prueba de chi-cuadrado de Pearson

**Tabla 2:** Comparación de los tiempos entre diagnóstico y modalidades de tratamiento entre pacientes con cáncer de mama incluidas o no en un programa de navegación (n = 359). Porto Alegre, RS, Brasil, 2023

Variable	Pacientes incluidas en el programa de navegación	Pacientes no incluidas en el programa	Total de pacientes	p-valor*
	Mediana + intervalos intercuartílicos	Mediana + intervalos intercuartílicos	Mediana + intervalos intercuartílicos	
	(días)	(días)	(días)	
	Mediana	Mediana	Mediana	
Tiempo entre diagnóstico e inicio de cualquier tratamiento (P25/75)	75,0 (50,0 / 98,0)	69,0 (42,0 / 112,0)	72,0 (0 / 271,0)	0,394
Tiempo entre diagnóstico y tratamiento neoadyuvante (P25/75)	72,0 (48,0 / 98,5)	56,0 (45,5 / 99,0)	71,5 (17,0 / 211,0)	0,273
Tiempo entre diagnóstico y tratamiento quirúrgico (P25/75)	231,0 (79,5 / 284,7)	79,0 (42,0 / 147,2)	94,0 (-19 / 652)	<0,001

*Prueba de Mann-Whitney

En relación con los tiempos diagnóstico-tratamiento en el grupo de pacientes atendidas por el SUS (Tabla 3), el tiempo mediano entre diagnóstico y cualquier tratamiento fue de 84 días. Cuando se consideró el factor navegación, las pacientes navegadas presentaron tiempos medianos entre diagnóstico y cualquier tratamiento (77 días) y tratamiento quimioterápico neoadyuvante (77 días), significativamente menores que los de aquellas no incluidas en el PN. No obstante, en relación con el tiempo entre diagnóstico e inicio del tratamiento quirúrgico, las pacientes navegadas presentaron un intervalo significativamente mayor que las no incluidas en el PN.

**Tabla 3:** Comparación de los tiempos entre diagnóstico y modalidades de tratamiento entre pacientes con cáncer de mama atendidas por el Sistema Único de Salud, incluidas o no en un programa de navegación (n = 153). Porto Alegre, RS, Brasil, 2023

Variable	Pacientes incluidas en el programa de navegación	Pacientes no incluidas en el programa	Total de pacientes	p-valor*
	Mediana + intervalos intercuartílicos	Mediana + intervalos intercuartílicos	Mediana + intervalos intercuartílicos	
	(días)	(días)	(días)	
	Mediana	Mediana	Mediana	
Tiempo entre diagnóstico e inicio de cualquier tratamiento (P25/75)	77,0 (55,0 / 100,0)	100,5 (73,2 / 150,2)	84,0 (60,0 / 122,0)	<0,001
Tiempo entre diagnóstico y tratamiento neoadyuvante (P25/75)	77,0 (53,0 / 99,7)	85,5 (50,7 / 112,2)	77,0 (52,7 / 101,0)	<0,001
Tiempo entre diagnóstico y tratamiento quirúrgico (P25/75)	236,0 (85,0 / 285,0)	128,0 (80,0 / 191,5)	152,0 (81,0 / 264,7)	<0,001

En el grupo de pacientes atendidas por el sistema de salud suplementaria (Tabla 4), el tiempo mediano entre diagnóstico y cualquier tratamiento fue de 42,5 días. Cuando se consideró la inclusión o no en el programa de navegación, las pacientes navegadas presentaron tiempos entre diagnóstico e inicio de cualquier tratamiento (38 días), diagnóstico e inicio de quimioterapia neoadyuvante (31 días), significativamente menores que los de las pacientes no navegadas, que presentaron intervalos de 47 y 50 días, respectivamente.

**Tabla 4:** Comparación de los tiempos entre diagnóstico y modalidades de tratamiento entre pacientes con cáncer de mama atendidas por la salud suplementaria, incluidas o no en un programa de navegación (n = 206). Porto Alegre, RS, Brasil, 2023

Variable	Pacientes incluidas en el programa de navegación	Pacientes no incluidas en el programa	Total de pacientes	p-valor*
	Mediana + intervalos intercuartílicos	Mediana + intervalos intercuartílicos	Mediana + intervalos intercuartílicos	
	(días)	(días)	(días)	
	Mediana	Mediana	Mediana	
Tiempo entre diagnóstico e inicio de cualquier tratamiento (P25/75)	38,0 (30,5 / 46,5)	47,0 (28,0 / 62,0)	42,5 (28,2 / 60,5)	<0,001
Tiempo entre diagnóstico y tratamiento neoadyuvante (P25/75)	31,0 (30,5 / 41,5)	50,0 (17,0 / 56,0)	35,5 (28,5 / 51,5)	<0,001
Tiempo entre diagnóstico y tratamiento quirúrgico (P25/75)	55,0 (44,0 / 264,0)	49,0 (29,0 / 81,0)	7,0 (52,5 / 101,0)	<0,001

*Prueba Mann-Whitney

En relación con los tiempos entre diagnóstico e inicio del tratamiento quirúrgico, la variable presentó un intervalo significativamente mayor en las pacientes navegadas (p<0,05). Cuando se considera la inclusión o no en el programa de navegación, segmentando por tipo de convenio, se observa que el tiempo entre diagnóstico y tratamiento quirúrgico continúa siendo mayor en este grupo.

## Discusión

Los resultados obtenidos en este estudio corroboran el perfil epidemiológico frecuentemente descrito en la literatura para la población con cáncer de mama. La media de edad de la muestra fue de 58,6±13,6 años y no hubo diferencia entre los grupos. El estudio que analizó el perfil epidemiológico de personas con diagnóstico de neoplasia de mama en Brasil evidenció que la incidencia de la enfermedad en el grupo etario de 50 a 59 años es predominante^4^. El mismo estudio también señaló que 98,7% de los casos ocurren en mujeres. De acuerdo con datos del INCA, el cáncer de mama femenino es predominante en todas las regiones de Brasil, y los casos de la enfermedad en hombres se consideran raros^4^.

El color de piel no blanca, la educación primaria incompleta y la atención por el SUS fueron más frecuentes en el grupo incluido en el PN. Factores socioeconómicos como desempleo, bajo nivel educativo, pobreza y color de piel/raza/etnia se encuentran entre los determinantes sociales más importantes de la salud, incluso en oncología^21^ ^-^ ^22^. Aspectos clínicos, características demográficas y otras desigualdades étnicas en el acceso a los servicios de salud han sido señalados como influyentes en el acceso al diagnóstico y al tratamiento oportuno del cáncer de mama en las mujeres^23^ ^-^ ^24^. Así, con el fin de reducir la disparidad de acceso, la navegación de pacientes se justifica, lo que se ve ratificado por los hallazgos de este estudio en lo que se refiere a los mejores indicadores de tiempo en las modalidades diagnóstico-cualquier tratamiento y diagnóstico-tratamiento neoadyuvante tanto entre pacientes atendidas por el SUS como por la salud suplementaria.

La navegación de pacientes tiene como premisa intervenir en las disparidades de acceso a la atención en salud y promover un tránsito oportuno de los individuos por el sistema de cuidados^13^ ^-^ ^14^ ^,^ ^24^. Puede inferirse que el grupo de pacientes navegadas, cuando se segmenta por modalidad/convenio de atención, especialmente en los tiempos diagnóstico-inicio de cualquier tratamiento y diagnóstico-inicio de tratamiento neoadyuvante, obtuvo un mejor manejo de las barreras para acceder al cuidado, dado el mejor desempeño en dichos indicadores. El abordaje realizado por el navegador proporciona a las pacientes apoyo y comunicación constante, ofreciendo informaciones fundamentales sobre el diagnóstico y la trayectoria terapéutica y ayudando a mantener una mejor adherencia al tratamiento^25^. Por lo tanto, este estudio se alinea con las premisas de la navegación de pacientes.

En relación con la estadificación al diagnóstico, 74,5% de la muestra presentó neoplasia en los estadios 0, I y II, sin diferencia entre los grupos. Entre las pacientes incluidas en el programa de navegación, se evidenció un volumen mayor de diagnósticos en estadio III y IV (35,3%) en comparación con las pacientes no incluidas (10,2%). Esto puede explicarse por el hecho de que las pacientes atendidas por el SUS correspondían mayoritariamente al grupo “navegadas”. En otras palabras, aunque la navegación haya mejorado el tiempo de acceso al tratamiento neoadyuvante y a cualquier tratamiento, las pacientes del sistema público siguieron tardando mucho más en acceder a los tratamientos que aquellas procedentes del sistema suplementario, lo que puede haber repercutido en una estadificación más avanzada del cáncer. A pesar de que esta realidad corrobora las inequidades sociales de Brasil, las pacientes del SUS que fueron incluidas en el PN tuvieron resultados de tiempo de acceso al tratamiento clínico significativamente mejores que aquellas que no lo fueron. Esto sugiere la necesidad de inversión por parte del gobierno en esta modalidad de actuación del enfermero, en especial en enfermedades progresivas.

El estudio transversal, analítico y retrospectivo realizado en 2024 en un hospital público de Brasilia identificó que 35% de las pacientes fueron diagnosticadas en estadio III, demostrando un diagnóstico más tardío en estas mujeres^26^. Otro estudio, realizado en Río de Janeiro, identificó una prevalencia de 43,7% de diagnósticos en estadio avanzado (estadios III y IV)^25^. Esta condición se observó entre mujeres con edad media de 49 años, color de piel no blanca, que vivían sin pareja y remitidas a través del SUS^27^. Aunque en menor proporción que en los estudios citados, nuestros hallazgos demuestran que existe margen de mejora en la reducción de diagnósticos en estadios III y IV, independientemente de la inclusión o no en el programa de navegación de pacientes.

El manejo del cáncer de mama requiere un abordaje personalizado, teniendo en cuenta, entre otros aspectos, la estadificación de la enfermedad, los resultados de la inmunohistoquímica y las dimensiones del tumor^28^. Actualmente, la intervención quirúrgica para la extirpación del tumor ya no es la única etapa crítica del tratamiento, sino una parte de un amplio proceso afrontado por las mujeres^28^ ^-^ ^29^. La quimioterapia, la radioterapia, la hormonoterapia y la inmunoterapia, por ejemplo, son formas complementarias de tratamiento que, a lo largo del tiempo, han contribuido de manera significativa a aumentar las tasas de supervivencia y el período libre de progresión de la enfermedad^29^. Considerando el número de modalidades de tratamiento realizadas, en el total de la muestra, 53,1% de las pacientes realizaron más de un tratamiento, sin diferencia significativa entre las pacientes navegadas y no navegadas. El dato sugiere que existe disponibilidad de diversas modalidades de tratamiento en el servicio estudiado, independientemente del apoyo o no proporcionado por el EN.

En relación con los tipos de tratamiento realizados, predominó el tratamiento quimioterápico (73,7%) entre las pacientes navegadas. Las pacientes en quimioterapia pueden presentar diversos efectos adversos y permanecer en tratamiento durante varios meses, dependiendo de la terapia indicada, lo que puede dificultar el seguimiento o incluso llevar al abandono del tratamiento^30^ ^-^ ^32^. Los signos y síntomas del tratamiento quimioterápico, como náuseas, vómitos, fatiga, mucositis, neutropenia febril, son algunos efectos secundarios que terminan contribuyendo a esta situación^30^ ^-^ ^32^. La adherencia al tratamiento, en este sentido, es un proceso multidimensional influido por aspectos sociales, económicos e individuales de la persona y requiere la ayuda de profesionales de la salud capacitados para el proceso educativo en salud^30^ ^-^ ^32^. De este modo, la coordinación del cuidado realizada por el EN se centra en garantizar una mejor adherencia al tratamiento y reducir las tasas de abandono debido a estos efectos secundarios.

Entre las pacientes no navegadas, predominó la realización de procedimientos quirúrgicos (92,5%). Esto puede atribuirse al hecho de que las pacientes que realizan únicamente esta modalidad de tratamiento no continúan el tratamiento clínico en el servicio y, por ello, no se incluyen en el PN, siendo uno de los criterios de inclusión en el PN realizar todo el tratamiento en el servicio. Otra hipótesis para este hallazgo es la mayor concentración de pacientes no navegadas en el grupo de salud suplementaria, el cual presentó menor tiempo de acceso al tratamiento quirúrgico y, en consecuencia, puede no haber requerido otra modalidad de atención además de la cirugía.

En Brasil, fue establecido por el *Ministério da Saúde* (MS), por medio de la Ley n.º 12.732/2012, un plazo de 60 días para el inicio del tratamiento de pacientes con diagnóstico de neoplasia maligna, contado a partir de la confirmación diagnóstica por informe de examen anatomopatológico hasta el primer día de realización de la modalidad terapéutica indicada^8^. Estudios señalan que este intervalo, cuando es superior a 60 días, puede asociarse a un peor pronóstico^33^ ^-^ ^34^. Un estudio realizado en Estados Unidos que evaluó el tiempo entre diagnóstico y tratamiento de mujeres de bajos ingresos con cáncer de mama señaló que cerca de 10% esperan un intervalo mayor o igual a 60 días para iniciar el tratamiento^33^. Este factor se asocia a una disminución de la supervivencia global a 5 años de 66% en este grupo^33^.

Los resultados del presente estudio evidencian que, aunque próximo a la recomendación legal^8^, el tiempo entre diagnóstico y cualquier tratamiento, y entre diagnóstico e inicio de tratamiento neoadyuvante, en el servicio investigado aún demanda acciones para mejorar dicho intervalo, principalmente en las pacientes del sistema público de salud. Esta realidad reafirma que el SUS demanda inversión significativa y permanente, así como refuerza una mirada crítica hacia la dimensión de su premisa de integralidad, incluyendo la optimización del tiempo de acceso. Conforme atestiguan los resultados aquí descritos, la actuación del EN puede ser una estrategia prometedora para este fin.

En el análisis de la variable tiempo entre diagnóstico e inicio del tratamiento quirúrgico, se evidenció que, principalmente, las pacientes atendidas por el SUS presentaron un intervalo prolongado para su realización. Tal hallazgo puede estar asociado al hecho de que, en el análisis de esta variable, no se consideraron únicamente los procedimientos quirúrgicos como primer tratamiento, sino que se incluyeron también los procedimientos realizados tras la finalización del tratamiento sistémico neoadyuvante (quimioterapia u hormonoterapia). Esto se explica porque, en el momento de la inclusión de la fecha de realización de la cirugía en la base de datos, no se especifica si esta fue anterior o posterior al tratamiento sistémico neoadyuvante, lo que imposibilita dicha selección para la realización de un análisis más preciso.

La neoadyuvancia se refiere a la realización de un tratamiento sistémico antes del tratamiento considerado de mayor impacto curativo, habitualmente la cirugía^35^ ^-^ ^36^, y tiene una duración media, en el cáncer de mama, de 4 a 6 meses, dependiendo del protocolo terapéutico indicado^37^. Anteriormente, estaba reservada para pacientes con tumores localmente avanzados o inoperables y tenía el objetivo exclusivo de reducir el tamaño del tumor para permitir una cirugía menos extensa y mutilante^35^ ^-^ ^36^. Sin embargo, en la actualidad, un mayor número de pacientes con cáncer de mama en estadio inicial tiene indicación de este tipo de tratamiento^35^ ^-^ ^36^. Pacientes con estadificación inicial pueden ser candidatas a la indicación de neoadyuvancia por el potencial beneficio en variables predictivas y pronósticas. Además, este tipo de tratamiento ha proporcionado la oportunidad de realizar cirugías más conservadoras, con el fin de reducir la necesidad de mastectomías totales^35^. De esta forma, el intervalo prolongado hasta el tratamiento quirúrgico puede estar vinculado al volumen de cirugías posneoadyuvancia, considerando que 53% de las pacientes realizan más de un tratamiento y la estadificación al diagnóstico, dado que un porcentaje significativamente mayor de pacientes de la muestra (89,8%) presentaba estadios iniciales al diagnóstico.

Al analizar el grupo general de pacientes, sin segmentación por convenio, se observó que no hubo diferencia significativa entre los indicadores de tiempo de acceso medidos. Las pacientes navegadas presentaron un tiempo mayor de acceso a los tratamientos. Sin embargo, es importante destacar que la variable tipo de convenio influyó en este hallazgo, dado que los indicadores de tiempo diagnóstico-tratamiento de las mujeres atendidas en la salud suplementaria son mucho menores que los de aquellas atendidas en el SUS, independientemente de si fueron asistidas o no por el Enfermero Navegador. Esto ratifica que las inequidades sociales son un marcador en el acceso al cuidado del cáncer de mama^4^ ^-^ ^6^ y, considerando los resultados segmentados por tipo de convenio, la navegación en enfermería puede constituir una estrategia provechosa para enfrentar dichas inequidades.

Los hallazgos relacionados con los tiempos diagnóstico-tratamiento refuerzan la preocupante disparidad, principalmente entre las pacientes atendidas por el SUS, dado que ese segmento de la muestra contribuyó al aumento en el tiempo de acceso al cuidado. Ante ello, es fundamental garantizar que las pacientes del sistema público de salud tengan acceso a programas de rastreo y diagnóstico precoz del cáncer, posibilitando un contacto más ágil con el EN, lo que podrá impactar positivamente en el tiempo de acceso al cuidado.

Un estudio brasileño, realizado con base en el análisis de los datos recopilados en el DATASUS y PAINEL-Oncología, evidenció que 43,6% de los individuos diagnosticados con neoplasia de mama por el SUS presentaron intervalos entre diagnóstico y tratamiento superiores a 60 días. Además, solo 21,2% iniciaron tratamiento entre 31 y 60 días^37^. La investigación transversal realizada en Piauí señaló que, entre los años 2016 y 2017, 71% de las mujeres diagnosticadas con cáncer de mama presentaron un intervalo mediano de 83,8 días para iniciar el tratamiento oncológico indicado^38^. Los autores atribuyeron dicho retraso, principalmente, a una desarticulación de la red de atención en oncología y sugirieron que existe necesidad de coordinar los flujos y organizar los puntos de atención especializada^38^. El enfermero se destaca en este proceso, dado que, por naturaleza, promueve la gestión del cuidado, lo que puede contribuir a la articulación de la asistencia en los puntos de atención de la red de salud.

La cohorte retrospectiva realizada en Minas Gerais evidenció un tiempo mediano de 71,5 días entre diagnóstico y tratamiento en mujeres tratadas en el sistema público y señaló que factores relacionados con las desigualdades sociales pueden influir en el acceso a los cuidados en el cáncer de mama^39^. Los hallazgos de esta investigación demuestran consonancia con lo señalado por la literatura.

En relación con las pacientes del sistema suplementario, también se evidenció que las pacientes navegadas presentaron medianas de tiempo diagnóstico-cualquier tratamiento y diagnóstico-tratamiento neoadyuvante significativamente menores que las pacientes no navegadas. Esto es interesante y demuestra que la navegación es provechosa independientemente del convenio de atención. En el caso de la salud suplementaria, además de mejorar desenlaces clínicos, el EN puede contribuir a la rentabilidad y sostenibilidad financiera de los servicios, una vez que el tiempo oportuno es un factor con implicaciones en este aspecto.

Un relato de experiencia describió la actuación de EN en la atención a pacientes con cáncer de mama en un hospital privado de un municipio del sur del país y demostró que, mediante las acciones realizadas por las profesionales, fue posible garantizar que las pacientes incluidas en el PN del servicio recibieran su tratamiento en tiempo oportuno^40^. Además, la revisión sistemática liderada por investigadores coreanos indicó que los estudios (n=6) incluidos evidenciaron que los enfermeros navegadores redujeron el número de días de intervalo entre diagnóstico y tratamiento en cerca de 17,5 días^41^. Tales evidencias agregan contundencia a los hallazgos de este estudio, confirmando que el EN, mediante la coordinación del cuidado, la mitigación de barreras de acceso y las orientaciones sobre el diagnóstico y la trayectoria de tratamiento, es capaz de mejorar el acceso en salud, incluso en situaciones en las que el tiempo es tan importante como en el cáncer.

Otro estudio publicado recientemente demostró que, tras la implementación de un programa de navegación para pacientes con cáncer de mama en el sur del país, fue posible reducir el tiempo entre diagnóstico e inicio de tratamiento neoadyuvante en cerca de 26%^42^. Los intervalos de tiempo (diagnóstico-tratamiento neoadyuvante y diagnóstico-cualquier tratamiento) significativamente más cortos presentados por las pacientes incluidas en el PN en comparación con las no incluidas alcanzaron una diferencia de 23 días en aquellas tratadas en el sistema público y 29 días en el sistema privado. Los retrasos para el inicio del tratamiento oncológico se conocen por comprometer seriamente los desenlaces clínicos. Demoras prolongadas en el intervalo diagnóstico-tratamiento se asocian directamente a una menor supervivencia de las pacientes con cáncer de mama^43^, lo que justifica que deben realizarse esfuerzos para mitigar y resolver barreras que puedan interferir en este indicador. Esto demuestra que los hallazgos corroboran lo que ha sido evidenciado en publicaciones científicas, comprobando que, mediante la navegación en enfermería, es posible impactar de forma positiva en los tiempos considerados críticos para la obtención de los mejores desenlaces clínicos.

Es prudente asumir como limitaciones de este estudio la ausencia de manejo de otras variables potencialmente confusoras además de la modalidad de convenio en la atención, así como la limitación natural de posible inconsistencia de registros. Se recomiendan altamente análisis más robustos que consideren otros desenlaces además del tiempo de acceso al tratamiento. No obstante, el estudio es relevante y confiere legitimidad científica para sustentar la difusión de la navegación de pacientes coordinada por enfermeros.

Este estudio aporta una contribución relevante al avance del conocimiento al demostrar, por medio de la comparación de los tiempos diagnóstico-tratamiento, la potencial efectividad de un programa de navegación para pacientes con cáncer de mama. Así, esta investigación destaca la importancia de la navegación como una estrategia capaz de optimizar el acceso a la atención oncológica, minimizar barreras del cuidado en el sistema de salud y potencialmente mejorar los desenlaces clínicos. Los datos presentados pueden, potencialmente, apoyar la implementación de políticas de salud centradas en reducir retrasos en el tratamiento oncológico.

## Conclusión

Las pacientes con cáncer de mama incluidas en un programa de navegación liderado por un enfermero tuvieron indicadores de tiempo de acceso al tratamiento en general y al tratamiento quimioterápico neoadyuvante significativamente menores que las pacientes no incluidas. Esto se evidenció principalmente cuando los grupos de pacientes navegadas y no navegadas se segmentaron por tipo de convenio (público o privado) en la atención. En ambos casos, las pacientes navegadas tuvieron tiempos significativamente mejores, aunque las pacientes procedentes del sistema público aún tardan considerablemente más en acceder al cuidado.

Estrategias desarrolladas por el EN en el PN, tales como la evaluación de las barreras de acceso a la atención, la elaboración e implementación de un plan de cuidados individualizado y la realización de acciones de educación en salud dirigidas a pacientes y familiares en relación con el diagnóstico y la trayectoria de tratamiento, pueden contribuir de forma positiva en estos intervalos críticos. Por lo tanto, además de evidenciar la efectividad del trabajo del Enfermero Navegador, este estudio atestigua la necesidad de invertir en la implementación de la navegación en los servicios de salud y en programas de rastreo y diagnóstico precoz del cáncer de mama en el SUS. Es importante señalar que deben realizarse futuras investigaciones, especialmente estudios prospectivos y estudios controlados, para evaluar desenlaces más amplios, como supervivencia, calidad de la adherencia terapéutica y calidad de vida, a fin de fortalecer la evidencia sobre la efectividad de la navegación en enfermería en el cuidado oncológico.

## Data Availability

Todos los datos generados o analizados durante este estudio están incluidos en este artículo publicado.
